# Homogeneous and isotropic cosmology in general teleparallel gravity

**DOI:** 10.1140/epjc/s10052-023-11462-6

**Published:** 2023-04-21

**Authors:** Lavinia Heisenberg, Manuel Hohmann, Simon Kuhn

**Affiliations:** 1 Institut für Theoretische Physik, Philosophenweg 16, 69120 Heidelberg, Germany; 2 Institute for Theoretical Physics, ETH Zurich, Wolfgang-Pauli-Strasse 27, 8093 Zurich, Switzerland; 3 Perimeter Institute, 31 Caroline Street N, Waterloo, ON Canada; 4 Laboratory of Theoretical Physics, Institute of Physics, University of Tartu, W. Ostwaldi 1, 50411 Tartu, Estonia

## Abstract

We derive the most general homogeneous and isotropic teleparallel geometries, defined by a metric and a flat, affine connection. We find that there are five branches of connection solutions, which are connected via several limits, and can further be restricted to the torsion-free and metric-compatible cases. We apply our results to several classes of general teleparallel gravity theories and derive their cosmological dynamics for all five branches. Our results show that for large subclasses of these theories the dynamics reduce to that of closely related metric or symmetric teleparallel gravity theories, while for other subclasses up to two new scalar degrees of freedom participate in the cosmological dynamics.

## Motivation

Various cosmological observations, such as the tension between late-time and early-time measurements of the Hubble parameter [1], show indications towards physics beyond the so-called $\Lambda$CDM model, which describes the dynamics of the universe through general relativity, a cosmological constant $\Lambda$ and cold dark matter (CDM), and has become widely accepted as the standard model of cosmology. In order to explain these observations, besides introducing new and unknown types of matter, numerous modified gravity theories have been developed and their cosmological dynamics has been studied [2–4]. Most conventionally, these theories depart from the common formulation of general relativity using the metric and its Levi-Civita connection, thereby attributing gravity to the curvature of the latter. However, besides these also a large number of so-called teleparallel theories exists, which attribute gravity to the torsion or nonmetricity of a flat connection, and these three possibilities have been subsumed under the title “geometric trinity of gravity” [5]. Further, instead of restricting the teleparallel geometry to being either metric-compatible or symmetric, one may also consider geometries which allow for both torsion and nonmetricity, leading to the class of general teleparallel gravity theories [6, 7]. Several classes of such theories have been proposed and studied [8–10].

In order to study the cosmological dynamics of any given gravity theory, one usually assumes that all dynamical fields present in the theory exhibit cosmological symmetry, i.e., homogeneity and isotropy, and are thus invariant under spatial rotations and translations. For the metric, this leads to the well-known Friedmann–Lemaître–Robertson–Walker form, while, e.g., a scalar field is assumed to be constant along hypersurfaces of constant time. For teleparallel gravity theories, one of the dynamical fields is a flat, affine connection, and so one must also impose homogeneity and isotropy on this field for consistency. Previous works in this direction have shown that the most general homogeneous and isotropic affine connection is described by five functions of time, two of which can be associated with torsion, while three are related to nonmetricity [11, 12]. If one imposes vanishing curvature and torsion, one finds three branches of solutions described by one function of time [13, 14], while for vanishing curvature and nonmetricity one finds three branches without any additional free functions besides those determining the metric [15].

The aim of this article is to continue the aforementioned line of studies and determine the most general homogeneous and isotropic teleparallel geometry, defined by a metric and a flat, affine connection, where the latter is restricted only by the condition of vanishing curvature, but may possess both torsion and nonmetricity, and to study the physical consequences of our findings in the context of different general teleparallel gravity theories. Moreover, we study how a coupling between matter and the teleparallel connection, which gives rise to a hypermomentum described as a cosmological hyperfluid [16], enters as a source into the cosmological field equations.

The outline of this article is as follows. In Sect. 2 we give a brief review of general teleparallel gravity, its dynamical fields and the general structure of the field equations. Bianchi identities are discussed in Sect. 3. We then construct the most general class of homogeneous and isotropic geometries in Sect. 4. In Sect. 5, we apply our findings to the matter side of the field equations and study the possible energy–momentum–hypermomentum sources. Further, in Sect. 6 we discuss several classes of general teleparallel gravity theories, derive their cosmological dynamics and study their general properties as well as relate them to previously studied classes of theories. Finally, we give an example for a possible contribution of the newly introduced connection degrees of freedom to the cosmological dynamics in Sect. 7. We end with a conclusion in Sect. 8.

## General teleparallel gravity

We start by giving a brief review of the class of general teleparallel gravity theories. In their metric-affine formulation, the dynamical fields are given by a Lorentzian metric $g_{\mu \nu }$ and an affine connection with coefficients $\Gamma ^{\mu }{}_{\nu \rho }$, which is imposed to be flat,

1 $$\begin{aligned} R^{\rho }{}_{\sigma \mu \nu } {=} \partial _{\mu }\Gamma ^{\rho }{}_{\sigma \nu } {-} \partial _{\nu }\Gamma ^{\rho }{}_{\sigma \mu } {+} \Gamma ^{\rho }{}_{\lambda \mu }\Gamma ^{\lambda }{}_{\sigma \nu } {-} \Gamma ^{\rho }{}_{\lambda \nu }\Gamma ^{\lambda }{}_{\sigma \mu } = 0.\nonumber \\ \end{aligned}$$

Note that this connection is different from the Levi-Civita connection, whose coefficients are the Christoffel symbols2where we use an empty circle to denote any quantity related to the Levi-Civita connection. Their difference can be written as3where the distortion $M^{\mu }{}_{\nu \rho }$ is composed of the contortion

4 $$\begin{aligned} K^{\mu }{}_{\nu \rho } = \frac{1}{2}\left( T_{\nu }{}^{\mu }{}_{\rho } + T_{\rho }{}^{\mu }{}_{\nu } - T^{\mu }{}_{\nu \rho }\right) , \end{aligned}$$

as well as the disformation

5 $$\begin{aligned} L^{\mu }{}_{\nu \rho } = \frac{1}{2}\left( Q^{\mu }{}_{\nu \rho } - Q_{\nu }{}^{\mu }{}_{\rho } - Q_{\rho }{}^{\mu }{}_{\nu }\right) , \end{aligned}$$

and these are defined through the torsion

6 $$\begin{aligned} T^{\mu }{}_{\nu \rho } = \Gamma ^{\mu }{}_{\rho \nu } - \Gamma ^{\mu }{}_{\nu \rho }, \end{aligned}$$

and the nonmetricity

7 $$\begin{aligned} Q_{\mu \nu \rho } = \nabla _{\mu }g_{\nu \rho } = \partial _{\mu }g_{\nu \rho } - \Gamma ^{\sigma }{}_{\nu \mu }g_{\sigma \rho } - \Gamma ^{\sigma }{}_{\rho \mu }g_{\nu \sigma }. \end{aligned}$$

The dynamical fields, among which we also include an arbitrary set $\psi ^I$ of matter fields, constitute the action, which we write in the form

8 $$\begin{aligned} S[g, \Gamma , \psi ] = S_{\text {g}}[g, \Gamma ] + S_{\text {m}}[g, \Gamma , \psi ], \end{aligned}$$

where $S_{\text {g}}$ is the gravitational action, which defines the gravity theory under consideration, and $S_{\text {m}}$ is a generic matter action. It follows that the variation of the latter can be written in the form

9 $$\begin{aligned} \delta S_{\text {m}}{} & {} = \int _M\left( \frac{1}{2}\Theta ^{\mu \nu }\delta g_{\mu \nu } + H_{\mu }{}^{\nu \rho }\delta \Gamma ^{\mu }{}_{\nu \rho } + \Psi _I\delta \psi ^I\right) \nonumber \\{} & {} \quad \times \sqrt{-g}\textrm{d}^4x, \end{aligned}$$

where $\Psi _I = 0$ are the matter field equations, and we introduced the energy–momentum tensor $\Theta _{\mu \nu }$ and the hypermomentum $H_{\mu }{}^{\nu \rho }$. For the gravitational part of the action, we similarly write

10 $$\begin{aligned} \delta S_{\text {g}} = -\int _M\left( \frac{1}{2}W^{\mu \nu }\delta g_{\mu \nu } + Y_{\mu }{}^{\nu \rho }\delta \Gamma ^{\mu }{}_{\nu \rho }\right) \sqrt{-g}\textrm{d}^4x, \end{aligned}$$

where $W^{\mu \nu }$ and $Y_{\mu }{}^{\nu \rho }$ depend on the gravity theory under consideration. When performing the variation with respect to the connection, one must take into account its flatness (1), either by adding a Lagrange multiplier and keeping the variation $\delta \Gamma ^{\mu }{}_{\nu \rho }$ of the connection arbitrary, or by allowing only variations of the form

11 $$\begin{aligned} \delta \Gamma ^{\mu }{}_{\nu \rho } = \nabla _{\rho }\xi ^{\mu }{}_{\nu } \end{aligned}$$

with a tensor field $\xi ^{\mu }{}_{\nu }$, which preserve the flatness by construction. Following either approach yields the connection field equations [10, 17]

12 $$\begin{aligned} \nabla _{\tau }Y_{\mu }{}^{\nu \tau } - M^{\omega }{}_{\tau \omega }Y_{\mu }{}^{\nu \tau } = \nabla _{\tau }H_{\mu }{}^{\nu \tau } - M^{\omega }{}_{\tau \omega }H_{\mu }{}^{\nu \tau }, \end{aligned}$$

while variation with respect to the metric yields the field equations

13 $$\begin{aligned} W_{\mu \nu } = \Theta _{\mu \nu }. \end{aligned}$$

As mentioned before, the dependence of the terms $W_{\mu \nu }$ and $Y_{\mu }{}^{\nu \rho }$ on the dynamical fields depends on the choice of the gravitational action, and thus the theory under consideration. A simple example is the general teleparallel equivalent of general relativity (GTEGR), whose action reads [6]

14 $$\begin{aligned} S_{\text {g}} = -\frac{1}{2\kappa ^2}\int \textrm{d}^4x\sqrt{-g}G, \end{aligned}$$

where the scalar *G* in the action is defined as

15 $$\begin{aligned} G= & {} 2M^\mu {}_{\nu [\mu }M^{\nu \rho }{}_{\rho ]} = \bigg (\frac{1}{4}Q^{\mu \nu \rho }Q_{\mu \nu \rho }\nonumber \\{} & {} - \frac{1}{2}Q^{\mu \nu \rho }Q_{\rho \mu \nu } - \frac{1}{4}Q^{\rho \mu }{}_{\mu }Q_{\rho \nu }{}^{\nu }\nonumber \\{} & {} + \frac{1}{2}Q^{\mu }{}_{\mu \rho }Q^{\rho \nu }{}_{\nu } + \frac{1}{4}T^{\mu \nu \rho }T_{\mu \nu \rho } + \frac{1}{2}T^{\mu \nu \rho }T_{\rho \nu \mu }\nonumber \\{} & {} - T^{\mu }{}_{\rho \mu }T_{\nu }{}^{\rho \nu } + T^{\mu \nu \rho }Q_{\nu \rho \mu } - T^{\mu }{}_{\rho \mu }Q_{\rho \nu }{}^{\nu } \nonumber \\{} & {} +T^{\mu }{}_{\rho \mu }Q^{\nu }{}_{\nu \rho }\bigg ). \end{aligned}$$

This action has the property that it agrees with the Einstein–Hilbert action up to a boundary term *B*,16and so its field equations turn out to be identical to those of general relativity [10],17We will discuss a number of generalizations of GTEGR in Sect. 6.

## Bianchi identity of general teleparallel theories

In GR the famous Bianchi identity states that the Einstein tensor is automatically covariantly conserved, and so is the energy–momentum tensor of matter by the Einstein equation. Here we want to derive the Bianchi identity of a general teleparallel theory and look at the consequences for hypermomentum conservation.

Let us assume again we have an action $S[g,\Gamma ,\psi ]$ of metric, connection, and matter fields. This action can be either $S_g$, $S_m$, or their sum, where the first is of course independent of $\psi$. In each case the action is by construction invariant under coordinate transformations, i.e. its value will not change if we perform an infinitesimal coordinate transformation $x^\mu \rightarrow x^\mu +\zeta ^\mu$. But the integrand in the action will change by its Lie derivative along $\zeta ^\mu$, so we obtain

18 $$\begin{aligned} 0= & {} \delta _\zeta S[g,\Gamma ,\psi ]=\int _M\nonumber \\{} & {} {\times } \left( {-}\frac{1}{2}\mathcal {E}^{\mu \nu }\mathcal {L}_\zeta g_{\mu \nu }{+}\mathcal {Y}_\alpha {}^{\mu \nu }\mathcal {L}_\zeta \Gamma ^\alpha {}_{\mu \nu }{+}\mathcal {P}_I\mathcal {L}_\zeta \psi ^I\right) \nonumber \\{} & {} \sqrt{-g}\textrm{d}^4x. \end{aligned}$$

Let us also define

19 $$\begin{aligned} \mathcal {C}_\mu {}^\nu =\nabla _\tau \mathcal {Y}_\mu {}^{\nu \tau }-M^\omega {}_{\tau \omega }\mathcal {Y}_\mu {}^{\nu \tau }. \end{aligned}$$

If we take for *S* the full action then $\mathcal {E}_{\mu \nu }=0$, $\mathcal {C}_\mu {}^\nu =0$, and $\mathcal {P}_I=0$ will be the equations of motion of metric, connection, and matter fields, respectively. For either of the three choices of *S* we will have $\mathcal {P}_I=0$ either trivially or by the matter field equation of motion $\Psi _I=0$, so we can drop this term; otherwise we must consider concrete field theories to find the form of $\mathcal {L}_\zeta \psi ^I$. For the other two Lie derivatives we have20

21 $$\begin{aligned} \mathcal {L}_\zeta \Gamma ^\alpha {}_{\mu \nu }&=\zeta ^\lambda \partial _\lambda \Gamma ^\alpha {}_{\mu \nu }+\partial _\mu \zeta ^\lambda \Gamma ^\alpha {}_{\lambda \nu }\nonumber \\&\quad +\partial _\nu \zeta ^\lambda \Gamma ^\alpha {}_{\mu \lambda }-\partial _\lambda \zeta ^\alpha \Gamma ^\lambda {}_{\mu \nu }+\partial _\mu \partial _\nu \zeta ^\alpha \nonumber \\ {}&=R^\alpha {}_{\mu \lambda \nu }\zeta ^\lambda +\nabla _\nu \nabla _\mu \zeta ^\alpha +\nabla _\nu (T^\alpha {}_{\lambda \mu }\zeta ^\lambda )\ . \end{aligned}$$

For our flat connection the Riemann tensor vanishes, so we have2223where we integrated by parts.^1^ Since this must hold for any $\zeta ^\mu$ we obtain the Bianchi identity24or in other form25This identity trivially holds if we take for *S* the full action $S_g+S_m$, although one finds that if the metric equation alone is fulfilled we still have

26 $$\begin{aligned} \nabla _\mu \mathcal {C}_\nu {}^\mu -T^\alpha {}_{\nu \mu }\mathcal {C}_\alpha {}^\mu =0 \end{aligned}$$

automatically. In theories without torsion the connection equation of motion is actually just $(\nabla _\mu -M^\omega {}_{\mu \omega })\mathcal {C}_\nu {}^\mu =0$, which is by the Bianchi identity fulfilled automatically whenever the metric equations are. Hence they become obsolete by the Bianchi identity. If we consider only $S=S_g$ we have $\mathcal {E}^{\mu \nu }=W^{\mu \nu }$ and $\mathcal {Y}_\alpha {}^{\mu \nu }=Y_\alpha {}^{\mu \nu }$, and (24) becomes a non-trivial identity between the left side of the equations of motion. One can check that it holds for all examples given below. When using the matter action $S_m$ we have $\mathcal {E}^{\mu \nu }=\Theta ^{\mu \nu }$, and we assume the matter energy–momentum tensor to be covariantly conserved on its own, . We then find that hypermomentum must be covariantly conserved as well in the form

27 $$\begin{aligned} \nabla _\mu \mathcal {C}_\nu {}^\mu -T^\alpha {}_{\nu \mu }\mathcal {C}_\alpha {}^\mu =0\, \mathcal {C}_\mu {}^\nu =\nabla _\tau H_\mu {}^{\nu \tau }-M^\omega {}_{\tau \omega } H_\mu {}^{\nu \tau }.\nonumber \\ \end{aligned}$$

Lastly we consider the case when the gravitational action also depends on a scalar field $\Phi$, as we will discuss below. Then , and we find the Bianchi identity28where $\Psi$ is the equation of motion of $\Phi$.

## Homogeneous and isotropic cosmology

In this section we derive the most general teleparallel geometry which is compatible with the assumption of cosmological symmetry, following closely the approach used for the metric and symmetric teleparallel cases [13–15]. Using spherical coordinates $(t,r,\vartheta ,\varphi )$, the generating vector fields establishing cosmological symmetry are the three rotation generators

29a $$\begin{aligned} \varrho _1&= \sin \varphi \partial _{\vartheta } + \frac{\cos \varphi }{\tan \vartheta }\partial _{\varphi }\,, \end{aligned}$$

29b $$\begin{aligned} \varrho _2&= -\cos \varphi \partial _{\vartheta } + \frac{\sin \varphi }{\tan \vartheta }\partial _{\varphi }\,, \end{aligned}$$

29c $$\begin{aligned} \varrho _3&= -\partial _{\varphi }\,, \end{aligned}$$

as well as the three translation generators

30a $$\begin{aligned} \tau _1&= \chi \sin \vartheta \cos \varphi \partial _r + \frac{\chi }{r}\cos \vartheta \cos \varphi \partial _{\vartheta } - \frac{\chi \sin \varphi }{r\sin \vartheta }\partial _{\varphi }\,, \end{aligned}$$

30b $$\begin{aligned} \tau _2&= \chi \sin \vartheta \sin \varphi \partial _r + \frac{\chi }{r}\cos \vartheta \sin \varphi \partial _{\vartheta } + \frac{\chi \cos \varphi }{r\sin \vartheta }\partial _{\varphi }\,, \end{aligned}$$

30c $$\begin{aligned} \tau _3&= \chi \cos \vartheta \partial _r - \frac{\chi }{r}\sin \vartheta \partial _{\vartheta }\,. \end{aligned}$$

We then demand that both the metric $g_{\mu \nu }$ and the flat affine connection $\Gamma ^{\mu }{}_{\nu \rho }$, which constitute the dynamical fields in the teleparallel geometry, are invariant under the action of these vector fields. This means that their Lie derivatives [18]

31 $$\begin{aligned} (\mathcal {L}_{X}g)_{\mu \nu } = X^{\rho }\partial _{\rho }g_{\mu \nu } + \partial _{\mu }X^{\rho }g_{\rho \nu } + \partial _{\nu }X^{\rho }g_{\mu \rho } \end{aligned}$$

and

32 $$\begin{aligned} (\mathcal {L}_{X}\Gamma )^{\mu }{}_{\nu \rho }= & {} X^{\sigma }\partial _{\sigma }\Gamma ^{\mu }{}_{\nu \rho } - \partial _{\sigma }X^{\mu }\Gamma ^{\sigma }{}_{\nu \rho } + \partial _{\nu }X^{\sigma }\Gamma ^{\mu }{}_{\sigma \rho }\nonumber \\{} & {} + \partial _{\rho }X^{\sigma }\Gamma ^{\mu }{}_{\nu \sigma } + \partial _{\nu }\partial _{\rho }X^{\mu } \end{aligned}$$

must vanish, where we used the abbreviation $\chi {=} \sqrt{1 {-} u^2r^2}$, and $u^2 \in \mathbb {R}$ indicates the spatial curvature. The latter can take positive or negative values, so that *u* can be real or imaginary; this choice of the parameter will turn out to be more practical than the more common curvature parameter $k = u^2$. It is well known that the most general metric which satisfies the condition (31) is the Robertson-Walker metric, whose non-vanishing components are given by

33 $$\begin{aligned} g_{tt} {=} {-}N^2, \quad g_{rr} {=} \frac{A^2}{\chi ^2}, \quad g_{\vartheta \vartheta } {=} A^2r^2, \quad g_{\varphi \varphi } {=} g_{\vartheta \vartheta }\sin ^2\vartheta , \end{aligned}$$

where we denote by $N = N(t)$ the lapse function and by $A = A(t)$ the scale factor. Note that we will keep the former general at this point, and make a convenient choice later in this article. Further, we introduce the hypersurface conormal $n_{\mu }$ and spatial metric $h_{\mu \nu }$, which allow us to decompose the metric in the form

34 $$\begin{aligned} g_{\mu \nu } = -n_{\mu }n_{\nu } + h_{\mu \nu }. \end{aligned}$$

Their non-vanishing components are given by

35 $$\begin{aligned} n_t {=} -N, \quad h_{rr} {=} \frac{A^2}{\chi ^2}, \quad h_{\vartheta \vartheta } {=} A^2r^2, \quad h_{\varphi \varphi } {=} h_{\vartheta \vartheta }\sin ^2\vartheta .\nonumber \\ \end{aligned}$$

Further, the Levi-Civita tensor $\epsilon _{\mu \nu \rho \sigma }$ of $g_{\mu \nu }$ gives rise to spatial Levi-Civita tensor $\varepsilon _{\mu \nu \rho }$ via

36 $$\begin{aligned} \varepsilon _{\mu \nu \rho } = n^{\sigma }\epsilon _{\sigma \mu \nu \rho }, \quad \epsilon _{\mu \nu \rho \sigma } = 4\varepsilon _{[\mu \nu \rho }n_{\sigma ]}. \end{aligned}$$

For the affine connection, we will proceed in two steps. In addition to the symmetry condition (32) for the six generating vector fields of cosmological symmetry, we must also impose the condition (1) of vanishing curvature. Starting with the former condition, one finds that the non-vanishing components of the most general cosmologically symmetric affine connection are given by [11, 12, 14]

37 $$\begin{aligned}{} & {} \Gamma ^t{}_{tt} = K_1, \quad \Gamma ^{\vartheta }{}_{r\vartheta } = \Gamma ^{\vartheta }{}_{\vartheta r} = \Gamma ^{\varphi }{}_{r\varphi } = \Gamma ^{\varphi }{}_{\varphi r} = \frac{1}{r}, \nonumber \\{} & {} \Gamma ^{\varphi }{}_{\vartheta \varphi } = \Gamma ^{\varphi }{}_{\varphi \vartheta } = \cot \vartheta , \quad \Gamma ^{\vartheta }{}_{\varphi \varphi } = -\sin \vartheta \cos \vartheta ,\nonumber \\{} & {} \Gamma ^t{}_{rr} = \frac{K_2}{\chi ^2}, \quad \Gamma ^r{}_{\vartheta \vartheta } = -r\chi ^2, \quad \Gamma ^r{}_{\varphi \varphi } = -r\chi ^2\sin ^2\vartheta , \quad \nonumber \\{} & {} \Gamma ^r{}_{\varphi \vartheta } = -\Gamma ^r{}_{\vartheta \varphi } = K_5r^2\chi \sin \vartheta ,\nonumber \\{} & {} \Gamma ^t{}_{\vartheta \vartheta } = K_2r^2, \quad \Gamma ^r{}_{tr} = \Gamma ^{\vartheta }{}_{t\vartheta } = \Gamma ^{\varphi }{}_{t\varphi }= K_3, \nonumber \\{} & {} \Gamma ^r{}_{rt} = \Gamma ^{\vartheta }{}_{\vartheta t} = \Gamma ^{\varphi }{}_{\varphi t} = K_4, \quad \Gamma ^r{}_{rr} = \frac{kr}{\chi ^2},\nonumber \\{} & {} \Gamma ^t{}_{\varphi \varphi } = K_2r^2\sin ^2\vartheta , \quad \Gamma ^{\vartheta }{}_{r\varphi } = -\Gamma ^{\vartheta }{}_{\varphi r} = \frac{K_5\sin \vartheta }{\chi }, \nonumber \\{} & {} \Gamma ^{\varphi }{}_{r\vartheta } = -\Gamma ^{\varphi }{}_{\vartheta r} = -\frac{K_5}{\chi \sin \vartheta }, \end{aligned}$$

where $K_1(t), \ldots , K_5(t)$ are functions of time. For this connection, we can now calculate the curvature tensor, which reads

38 $$\begin{aligned} R^{\mu }{}_{\nu \rho \sigma }= & {} 2\frac{K_3(K_4 - K_1) + \partial _tK_3}{N^2}n_{\nu }n_{[\rho }h^{\mu }_{\sigma ]}\nonumber \\{} & {} + 2\frac{K_2(K_4 - K_1) - \partial _tK_2}{A^2}n^{\mu }n_{[\rho }h_{\sigma ]\nu }\nonumber \\{} & {} + 2\frac{K_2K_5N}{A^3}n^{\mu }\varepsilon _{\nu \rho \sigma } - 2\frac{K_3K_5}{NA}n_{\nu }\varepsilon ^{\mu }{}_{\rho \sigma } \nonumber \\{} & {} - 2\frac{\partial _tK_5}{NA}\varepsilon ^{\mu }{}_{\nu [\rho }n_{\sigma ]} + 2\frac{u^2 + K_2K_3 - K_5^2}{A^2}h^{\mu }_{[\rho }h_{\sigma ]\nu },\nonumber \\ \end{aligned}$$

making use of the decomposition (34) we introduced before. Note that all terms appearing in this decomposition are independent, and so their coefficients must vanish separately. Hence, the connection is flat if and only if

39 $$\begin{aligned} \partial _tK_5= & {} K_2K_5 = K_3K_5 = u^2 + K_2K_3 - K_5^2 \nonumber \\= & {} K_3(K_4 - K_1)+ \partial _tK_3 = K_2(K_4 - K_1)\nonumber \\{} & {} - \partial _tK_2 = 0. \end{aligned}$$

In order to determine the most general solution to these conditions, we distinguish the following two cases:

1. We start by assuming $u = 0$, i.e., vanishing spatial curvature. In this case we have the condition $K_2K_3 = K_5^2$, so either both sides are vanishing or non-vanishing. However, from $K_2K_5 = K_3K_5 = 0$ follows that $K_5 = 0$ or $K_2 = K_3 = 0$. Hence, the only option is $K_5 = K_2K_3 = 0$. Therefore, at least one of $K_2$ or $K_3$ must vanish, which leaves three cases to be distinguished:
  - Setting $K_2 = K_3 = 0$, the remaining equations are already satisfied and no further restrictions on the parameter functions arise. $K_1$ and $K_4$ are the only parameters left, which are arbitrary and unconstrained.
  - For $K_3 = 0$ and $K_2 \ne 0$, $K_2$ is a free function. Now only one of $K_1$ and $K_4$ is left undetermined, since the difference is constrained to satisfy
    40 $$\begin{aligned} K_4 - K_1 = \frac{\partial _tK_2}{K_2}. \end{aligned}$$
  - Assuming $K_2 = 0$ and $K_3 \ne 0$, one obtains a similar result as in the previous case, but now $K_3$ is a free function and the difference between $K_1$ and $K_4$ must satisfy
    41 $$\begin{aligned} K_4 - K_1 = -\frac{\partial _tK_3}{K_3}. \end{aligned}$$
2. We then continue with the spatially curved case $u \ne 0$. Now we can further distinguish the following two cases:
  - $K_5 \ne 0$: From $K_2K_5 = K_3K_5 = 0$ follows $K_2 = K_3 = 0$. Hence, $K_5 = \pm u$, which becomes imaginary for negative curvature $u^2<0$, and the remaining equations are satisfied. $K_1$ and $K_4$ are left undetermined.
  - $K_5 = 0$: In this case one has $K_2K_3 = -u^2 \ne 0$ and so both must be non-zero and inversely proportional, so that only one of them can be chosen arbitrarily. This further implies
    42 $$\begin{aligned} 0 = \partial _tK_2K_3 + K_2\partial _tK_3, \end{aligned}$$
    and so
    43 $$\begin{aligned} K_4 - K_1 = \frac{\partial _tK_2}{K_2} = -\frac{\partial _tK_3}{K_3}, \end{aligned}$$
    so that the remaining equations consistently determine the difference between $K_1$ and $K_4$.

We see that there are five different branches, in each of which the flat, cosmologically symmetric connection is determined by two functions of time. In the following, however, it will turn out to be more convenient to introduce a different parametrization, which is based on the expressions

44 $$\begin{aligned} T^{\mu }{}_{\nu \rho }= & {} 2T_1h^{\mu }_{[\nu }n_{\rho ]} + 2T_2\varepsilon ^{\mu }{}_{\nu \rho }, \nonumber \\ Q_{\rho \mu \nu }= & {} 2Q_1n_{\rho }n_{\mu }n_{\nu } + 2Q_2n_{\rho }h_{\mu \nu } + 2Q_3h_{\rho (\mu }n_{\nu )}. \end{aligned}$$

for the most general cosmologically symmetric torsion and nonmetricity tensors [11, 16], where $T_1, T_2, Q_1, Q_2, Q_3$ are functions of time *t*, which serve as an alternative parametrization to the previously introduced $K_1, \ldots , K_5$. It follows from the tensor character of the expressions on both sides of the equations that these new quantities are scalars under coordinate transformations, which makes them more suitable as dynamical variables in the cosmological field equations. They are related to the previously introduced quantities by

45 $$\begin{aligned} T_1= & {} \frac{K_4 - K_3}{N}, \quad T_2 = \frac{K_5}{A}, \quad Q_1 = \frac{\partial _tN}{N^2} - \frac{K_1}{N}, \nonumber \\ Q_2= & {} \frac{1}{N}\left( K_4 - \frac{\partial _tA}{A}\right) , \quad Q_3 = \frac{K_3}{N} - \frac{K_2N}{A^2}. \end{aligned}$$

From the case distinction discussed above, we see that in all cases we are free to choose the function $K_4$, or equivalently, $Q_2$ in the scalar variables, so that we can use it as a dynamical variable in all branches, which we denote by $K = Q_2$. For the remaining branches of flat connections, we need to introduce different parametrizations, in order to express the remaining scalar variables in terms of one additional, independent, dynamical variable. We follow the same distinction as before, and use the Hubble parameter

46 $$\begin{aligned} H = \frac{\partial _tA}{NA} \end{aligned}$$

for convenience.

1. For $u = 0$, we have $K_5 = 0$, and therefore $T_2 = 0$. We then distinguish further:
  - In the branch $K_2 = K_3 = 0$, we find $Q_3 = 0$. We are free to choose $K_1$, and hence can define the second variable as $L = Q_1$. The remaining scalar quantity is determined as $T_1 = H + K$.
  - For $K_2 \ne 0$, one has $K_2$ as a free function, and so can use $L = Q_3$ as dynamical variable. $K_1$ is fixed, and so are
    47 $$\begin{aligned} T_1 = H + K, \quad Q_1 = -K + H + \frac{\partial _tL}{NL}. \end{aligned}$$
  - For $K_3 \ne 0$, one can freely choose $K_3$, which again allows to define $L = Q_3$ as dynamical variable. In this case the remaining scalars read
    48 $$\begin{aligned} T_1 = H + K - L, \quad Q_1 = -K - H - \frac{\partial _tL}{NL}. \end{aligned}$$
2. For the spatially curved case $u \ne 0$, we use the following two parametrizations:
  - For $K_5 \ne 0$, we choose the sign of *u* in the parametrization such that $K_5 = u$, and are free to choose $L = Q_1$. The other sign convention of *u* will give the same result with the replacement $u\rightarrow -u$. This yields
    49 $$\begin{aligned} T_1 = H + K, \quad T_2 = \frac{u}{A}, \quad Q_3 = 0. \end{aligned}$$
  - Finally, for $K_5 = 0$, we can choose $L=H+K-T_1$ such that
    50 $$\begin{aligned} T_1{} & {} = H + K - L, \quad T_2 = 0, \quad Q_1 = -K - H \nonumber \\{} & {} \quad - \frac{\partial _tL}{NL}, \quad Q_3 = L + \frac{u^2}{A^2L}. \end{aligned}$$

**Table 1:** Branches for flat connections with cosmological symmetry

	*u*	$K_2$	$K_3$	$\mathcal {T}_1$	$\mathcal {T}_2$	$\mathcal {Q}_1$	$\mathcal {Q}_2$	$\mathcal {Q}_3$
1a	$= 0$	$= 0$	$= 0$	$\mathcal {H} + \mathcal {K}$	0	$\mathcal {L}$	$\mathcal {K}$	0
1b	$= 0$	$\ne 0$	$= 0$	$\mathcal {H} + \mathcal {K}$	0	$-\mathcal {K} + \dfrac{\mathcal {L}'}{\mathcal {L}}$	$\mathcal {K}$	$\mathcal {L}$
1c	$= 0$	$= 0$	$\ne 0$	$\mathcal {H} + \mathcal {K} - \mathcal {L}$	0	$-\mathcal {K} - \dfrac{\mathcal {L}'}{\mathcal {L}}$	$\mathcal {K}$	$\mathcal {L}$
2a	$\ne 0$	$= 0$	$= 0$	$\mathcal {H} + \mathcal {K}$	*u*	$\mathcal {L}$	$\mathcal {K}$	0
2b	$\ne 0$	$\ne 0$	$\ne 0$	$\mathcal {H} + \mathcal {K} - \mathcal {L}$	0	$-\mathcal {K} - \dfrac{\mathcal {L}'}{\mathcal {L}}$	$\mathcal {K}$	$\mathcal {L} + \dfrac{u^2}{\mathcal {L}}$

To further simplify the parametrizations, it turns out to be convenient to introduce rescaled quantities

51 $$\begin{aligned}{} & {} \mathcal {H} = AH, \quad \mathcal {K} = AK, \quad \mathcal {L} = AL, \nonumber \\{} & {} \quad \mathcal {T}_i = AT_i, \quad \mathcal {Q}_i = AQ_i, \end{aligned}$$

where $\mathcal {H}$ is the conformal Hubble parameter, as well as the conformal time derivative

52 $$\begin{aligned} f' = \frac{A}{N}\partial _tf \end{aligned}$$

for any function *f* of time. In terms of these quantities, the parametrizations simplify, and take the form summarized in Table 1.

It is worth discussing how these different branches of cosmologies are related to each other. For the spatially curved branch 2a, we see that $K_2 = K_3 = 0$ everywhere, and so this property is preserved also in the limit $u \rightarrow 0$, leading to the branch 1a. This is different for the spatially curved branch 2b. Using the parametrization given in Table 1, the limit $u \rightarrow 0$ leads to the branch 1c. However, this limit depends on the choice of the parametrization. By introducing a new parameter function $\tilde{\mathcal {L}} = u^2/\mathcal {L}$, the parametrization of the branch 2b becomes

53 $$\begin{aligned} \mathcal {T}_1= & {} \mathcal {H} + \mathcal {K} - \frac{u^2}{\tilde{\mathcal {L}}}, \quad \mathcal {T}_2 = 0, \quad \mathcal {Q}_1 = -\mathcal {K}+ \frac{\tilde{\mathcal {L}}'}{\tilde{\mathcal {L}}}, \nonumber \\ \mathcal {Q}_2= & {} \mathcal {K}, \quad \mathcal {Q}_3 = \tilde{\mathcal {L}} + \frac{u^2}{\tilde{\mathcal {L}}}. \end{aligned}$$

Taking the limit $u \rightarrow 0$, and renaming $\tilde{\mathcal {L}}$ to $\mathcal {L}$, we obtain the branch 1b. Finally, defining a new parameter instead as $\tilde{\mathcal {L}} = \mathcal {L}/u$, one obtains

54 $$\begin{aligned}{} & {} \mathcal {T}_1 = \mathcal {H} + \mathcal {K} - u\tilde{\mathcal {L}}, \quad \mathcal {T}_2 = 0, \quad \mathcal {Q}_1 = -\mathcal {K} - \frac{\tilde{\mathcal {L}}'}{\tilde{\mathcal {L}}},\nonumber \\{} & {} \quad \mathcal {Q}_2 = \mathcal {K}, \quad \mathcal {Q}_3 = u\left( \tilde{\mathcal {L}} + \frac{1}{\tilde{\mathcal {L}}}\right) . \end{aligned}$$

In the limit $u \rightarrow 0$, we obtain the branch 1a, with the identification of the new parameter

55 $$\begin{aligned} \mathcal {L} = -\mathcal {K} - \frac{\tilde{\mathcal {L}}'}{\tilde{\mathcal {L}}}. \end{aligned}$$

**Fig. 1:**
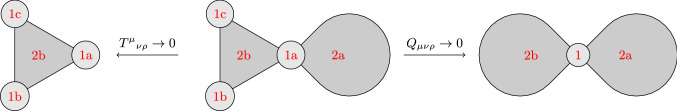
Schematic relation between different cosmologically symmetric branches

Further, one may consider the special cases of vanishing torsion and nonmetricity, respectively. First note that the branch 2a has explicit torsion $\mathcal {T}_2 = u$, and so does not have a limit with vanishing torsion. For the remaining branches, this limit is obtained by solving $\mathcal {T}_1 = 0$ for $\mathcal {K}$, and one obtains the four branches of symmetric teleparallel cosmology [13, 14]. Similarly, to obtain vanishing nonmetricity, one sets $\mathcal {K} = 0$ and then solves for $\mathcal {L}$. In this case the three spatially flat ($u = 0$) branches assume the common limit $\mathcal {T}_1 = \mathcal {H}$ and $\mathcal {T}_2 = 0$. Together with the two spatially curved branches, these agree with the result found for metric teleparallel cosmology [15]. These relations are illustrated in Fig. 1.

In the following sections, we will apply the conditions of homogeneity and isotropy to the matter source and gravitational field equations of teleparallel gravity theories.

## Cosmologically symmetric field equations and energy–momentum–hypermomentum

We apply the conditions of cosmological symmetry introduced in the previous section to the generic form (13) of the general teleparallel field equations. Assuming that the teleparallel geometry given by a metric and a flat affine connection is homogeneous and isotropic, it follows that also the left hand side of the gravitational field equations, which is constituted by these fields, has this symmetry, and must therefore be of the form

56 $$\begin{aligned}{} & {} W_{\mu \nu } = \mathfrak {N}n_{\mu }n_{\nu } + \mathfrak {H}h_{\mu \nu }, \quad \nonumber \\{} & {} \nabla _{\tau }Y_{\mu }{}^{\nu \tau }s - M^{\omega }{}_{\tau \omega }Y_{\mu }{}^{\nu \tau } = \mathfrak {T}n_{\mu }n^{\nu } + \mathfrak {S}h_{\mu }{}^{\nu }, \end{aligned}$$

where the dependence of the scalars $\mathfrak {N}, \mathfrak {H}, \mathfrak {T}, \mathfrak {S}$ follows from the gravity theory under consideration. For this form of hypermomentum the connection equation of motion becomes symmetric in $\mu$ and $\nu$. Hence, the right hand side of the field equations must be of the same form. For the energy–momentum tensor $\Theta _{\mu \nu }$, the cosmological symmetry condition leads to the perfect fluid form

57 $$\begin{aligned} \Theta _{\mu \nu } = \rho n_{\mu }n_{\nu } + ph_{\mu \nu }. \end{aligned}$$

Similarly, imposing the cosmological symmetry on the hypermomentum leads to the form [16]

58 $$\begin{aligned} H_{\rho \mu \nu }= & {} \phi h_{\mu \rho }n_{\nu } + \chi h_{\nu \rho }n_{\mu } + \psi h_{\mu \nu }n_{\rho } \nonumber \\{} & {} + \omega n_{\mu }n_{\nu }n_{\rho } - \zeta \varepsilon _{\rho \mu \nu }. \end{aligned}$$

Note that here and for the rest of the paper $\chi$ refers to the field appearing in the hypermomentum, and not to the expression $\sqrt{1-u^2r^2}$. Inserting these expressions into the gravitational field equations, one finds that they take the form

59 $$\begin{aligned} \mathfrak {N} = \rho , \quad \mathfrak {H} = p \end{aligned}$$

for the metric equations, while the connection equations read

60a $$\begin{aligned} -A\mathfrak {T}&= \omega ' + 3[\mathcal {H}\omega + (\mathcal {H} - \mathcal {T}_1 + \mathcal {Q}_2- \mathcal {Q}_3)\psi \nonumber \\&\quad + (\mathcal {H} - \mathcal {T}_1 + \mathcal {Q}_2)\chi ]\,, \end{aligned}$$

60b $$\begin{aligned} -A\mathfrak {S}&= \phi ' + 3\mathcal {H}\phi + (\mathcal {H} - \mathcal {T}_1 + \mathcal {Q}_2 - \mathcal {Q}_3)\psi \nonumber \\&\quad + (\mathcal {H} - \mathcal {T}_1 + \mathcal {Q}_2)\chi \,. \end{aligned}$$

Note that the latter depend on the choice of the cosmologically symmetric connection. For the five different branches we found, they are given as follows. For the spatially curved branch 2a and its flat limit 1a we find

61 $$\begin{aligned} -A\mathfrak {T}= & {} \omega ' + 3\mathcal {H}\omega , \nonumber \\ -A\mathfrak {S}= & {} \phi ' + 3\mathcal {H}\phi . \end{aligned}$$

The spatially curved branch 2b yields

62 $$\begin{aligned} -A\mathfrak {T}= & {} \omega ' + 3\mathcal {H}\omega + 3\mathcal {L}\chi - 3\frac{u^2}{\mathcal {L}}\psi , \nonumber \\ -A\mathfrak {S}= & {} \phi ' + 3\mathcal {H}\phi + \mathcal {L}\chi - \frac{u^2}{\mathcal {L}}\psi . \end{aligned}$$

For the spatially flat branch 1c one has

63 $$\begin{aligned} -A\mathfrak {T}= & {} \omega ' + 3\mathcal {H}\omega + 3\mathcal {L}\chi , \nonumber \\ -A\mathfrak {S}= & {} \phi ' + 3\mathcal {H}\phi + \mathcal {L}\chi , \end{aligned}$$

while the branch 1b yields

64 $$\begin{aligned} -A\mathfrak {T}= & {} \omega ' + 3\mathcal {H}\omega - 3\mathcal {L}\psi , \quad \nonumber \\ -A\mathfrak {S}= & {} \phi ' + 3\mathcal {H}\phi - \mathcal {L}\psi . \end{aligned}$$

We will make use of these expressions in the following sections, where we will derive the cosmological field equations for a number of example theories and discuss their physical implications.

## Application to teleparallel theories

We now apply our findings to a few classes of general teleparallel gravity theories and study their cosmological dynamics. In Sect. 6.1 we study the *f*(*G*) class of theories, whose Lagrangian is given by an arbitrary function *f* of the GTEGR Lagrangian *G*. Another class of theories based on the most general Lagrangian which is quadratic in torsion and nonmetricity is studied in Sect. 6.2. Finally, we consider a class of theories with a non-minimally coupled scalar field in Sect. 6.3.

### *f*(*G*) gravity

The first example we study is a generalization of GTEGR whose action is given by

65 $$\begin{aligned} S_{\text {g}} = -\frac{1}{2\kappa ^2}\int \textrm{d}^4x\sqrt{-g}f(G), \end{aligned}$$

where *G* denotes the scalar (15).66Varying the action with respect to the metric and the connection, one obtains the field equations [10] 67a67b One can then evaluate these for the cosmologically symmetric branches of teleparallel geometries. First note that the scalar *G* takes the cosmological value

68 $$\begin{aligned}&G = \frac{3}{A^2}[\mathcal {Q}_3(\mathcal {Q}_1 - \mathcal {Q}_2 + 2\mathcal {T}_1) + 2(\mathcal {Q}_2 - \mathcal {T}_1)^2 - 2\mathcal {T}_2^2]. \end{aligned}$$

The structure of the resulting cosmological field equations depends on the choice of the branch.

We start with the spatially curved branch 2a, for which the metric field equations become

69a $$\begin{aligned}&12\mathcal {H}^2\frac{f'}{A^2} - f = 2\kappa ^2\rho \,, \end{aligned}$$

69b $$\begin{aligned}&-4\mathcal {E}\frac{f''}{A^4} - 4(\mathcal {H}' + 2\mathcal {H}^2 - u^2)\frac{f'}{A^2} + f = 2\kappa ^2p\,, \end{aligned}$$

with

70 $$\begin{aligned} \mathcal {E}=A^2\mathcal {H}G', \end{aligned}$$

while the left hand side of the connection equations vanishes identically, so that they reduce to the continuity equations

71 $$\begin{aligned} \omega ' + 3\mathcal {H}\omega = \phi ' + 3\mathcal {H}\phi = 0. \end{aligned}$$

It is remarkable that the two functions $\mathcal {K}$ and $\mathcal {L}$ describing the dynamics of the flat connection do not explicitly appear in these equations. Calculating

72 $$\begin{aligned} G = \frac{6(\mathcal {H}^2 - u^2)}{A^2}, \end{aligned}$$

we see that these do not enter implicitly through *f* either, and so fully decouple for this branch. These findings also hold in the limit $u \rightarrow 0$, in which the branch 1a is obtained, for which the cosmological field equations become

73a $$\begin{aligned} 12\mathcal {H}^2\frac{f'}{A^2} - f&= 2\kappa ^2\rho \,, \end{aligned}$$

73b $$\begin{aligned} -4\mathcal {E}\frac{f''}{A^4} - 4(\mathcal {H}' + 2\mathcal {H}^2)\frac{f'}{A^2} + f&= 2\kappa ^2p\,, \end{aligned}$$

and the scalar *G* becomes

74 $$\begin{aligned} G = 6\frac{\mathcal {H}^2}{A^2}, \end{aligned}$$

and (70) still holds. Note that these equations agree with those of the metric teleparallel class of *f*(*T*) theories; while the spatially curved case (69) yield those for the “axial” branch of metric teleparallel cosmologies, the flat case (73) yields its flat limit [15, 19].

The situation is similar, yet different for the remaining branches. For the spatially curved case 2b, the metric cosmological field equations become

75a $$\begin{aligned}&-9(u^2 + \mathcal {L}^2)\mathcal {E}\frac{f''}{A^4\mathcal {L}^4} \nonumber \\&\quad + \left[ 6\mathcal {H}\mathcal {L}(u^2 + 2\mathcal {H}\mathcal {L} - \mathcal {L}^2) - 3(u^2 + \mathcal {L}^2)\mathcal {L}'\right] \nonumber \\&\quad \frac{f'}{A^2\mathcal {L}^2} - f = 2\kappa ^2\rho \,, \end{aligned}$$

75b $$\begin{aligned}&3(u^2 + 4\mathcal {H}\mathcal {L} - 3\mathcal {L}^2)\mathcal {E}\frac{f''}{A^4\mathcal {L}^4} \nonumber \\&\quad + \left\{ 2\mathcal {L}[2\mathcal {L}(u^2 - 2\mathcal {H}^2) - 3\mathcal {H}(u^2 - \mathcal {L}^2)] - 4\mathcal {L}^2\mathcal {H}' \right. \nonumber \\&\quad \left. +3(u^2 + \mathcal {L}^2)\mathcal {L}'\right\} \frac{f'}{A^2\mathcal {L}^2} + f \nonumber \\&\quad = 2\kappa ^2p\,, \end{aligned}$$

while the connection equations become

76a $$\begin{aligned}&-9(u^2 + \mathcal {L}^2)\mathcal {E}\frac{f''}{A^4\mathcal {L}^4} = \frac{2\kappa ^2}{A}\nonumber \\&\quad \left( \omega ' + 3\mathcal {H}\omega + 3\mathcal {L}\chi - 3\frac{u^2}{\mathcal {L}}\psi \right) \,, \end{aligned}$$

76b $$\begin{aligned}&-3(u^2 + \mathcal {L}^2)\mathcal {E}\frac{f''}{A^4\mathcal {L}^4} = \frac{2\kappa ^2}{A}\left( \phi ' + 3\mathcal {H}\phi + \mathcal {L}\chi - \frac{u^2}{\mathcal {L}}\psi \right) \,, \end{aligned}$$

where

77 $$\begin{aligned} \mathcal {E}= & {} 4\mathcal {H}\mathcal {L}^2(u^2 + \mathcal {H}\mathcal {L})(\mathcal {H} - \mathcal {L}) - 2\mathcal {L}^2(u^2 + 2\mathcal {H}\mathcal {L}\mathcal {L}^2)\mathcal {H}' \nonumber \\{} & {} - 2u^2\mathcal {L}'^2 + \mathcal {L}(u^2 + \mathcal {L}^2)\mathcal {L}'' = -A^2\mathcal {L}^3\frac{G'}{3}, \end{aligned}$$

with

78 $$\begin{aligned}&G = \frac{3}{\mathcal {L}^2A^2}[2\mathcal {L}(\mathcal {H} - \mathcal {L})(u^2 + \mathcal {H}\mathcal {L}) - (u^2 + \mathcal {L}^2)\mathcal {L}']. \end{aligned}$$

We now see that the scalar $\mathcal {L}$ enters the field equations, while $\mathcal {K}$ does not. Note that the connection field equations can be satisfied only if the hypermomentum satisfies the combined conservation equation

79 $$\begin{aligned} (\omega + 3\phi )' + 3\mathcal {H}(\omega + 3\phi ) = 0. \end{aligned}$$

This holds also for the flat limiting cases; for the case 1c we find the equations

80a $$\begin{aligned}&-9\mathcal {L}\mathcal {E}\frac{f''}{A^4}+ 3(4\mathcal {H}^2 - 2\mathcal {H}\mathcal {L} - \mathcal {L}')\frac{f'}{A^2} - f = 2\kappa ^2\rho \,, \end{aligned}$$

80b $$\begin{aligned}&3(4\mathcal {H} - 3\mathcal {L})\mathcal {E}\frac{f''}{A^4} - (4\mathcal {H}' - 3\mathcal {L}' - 6\mathcal {H}\mathcal {L} + 8\mathcal {H}^2)\frac{f'}{A^2} \nonumber \\&\qquad + f = 2\kappa ^2p \end{aligned}$$

80c $$\begin{aligned}&-9\mathcal {L}\mathcal {E}\frac{f''}{A^4} = \frac{2\kappa ^2}{A}(\omega ' + 3\mathcal {H}\omega + 3\mathcal {L}\chi )\,, \end{aligned}$$

80d $$\begin{aligned}&-3\mathcal {L}\mathcal {E}\frac{f''}{A^4} = \frac{2\kappa ^2}{A}(\phi ' + 3\mathcal {H}\phi + \mathcal {L}\chi )\,, \end{aligned}$$

with

81 $$\begin{aligned} \mathcal {E} = 4\mathcal {H}^2(\mathcal {H} - \mathcal {L}) - 2(2\mathcal {H} - \mathcal {L})\mathcal {H}' + \mathcal {L}'' = -A^2\frac{G'}{3}, \end{aligned}$$

and

82 $$\begin{aligned} G = \frac{6\mathcal {H}(\mathcal {H} - \mathcal {L}) - 3\mathcal {L}'}{A^2}, \end{aligned}$$

while for the branch 1b we have

83a $$\begin{aligned}&-9\mathcal {L}\mathcal {E}\frac{f''}{A^4} + 3(4\mathcal {H}^2 + 2\mathcal {H}\mathcal {L} + \mathcal {L}')\frac{f'}{A^2} - f = 2\kappa ^2\rho \,, \end{aligned}$$

83b $$\begin{aligned}&3(4\mathcal {H} + \mathcal {L})\mathcal {E}\frac{f''}{A^4} - (4\mathcal {H}' + 3\mathcal {L}' + 6\mathcal {H}\mathcal {L} + 8\mathcal {H}^2)\nonumber \\&\quad \frac{f'}{A^2} + f = 2\kappa ^2p \end{aligned}$$

83c $$\begin{aligned}&-9\mathcal {L}\mathcal {E}\frac{f''}{A^4} = \frac{2\kappa ^2}{A}(\omega ' + 3\mathcal {H}\omega - 3\mathcal {L}\psi )\,, \end{aligned}$$

83d $$\begin{aligned}&-3\mathcal {L}\mathcal {E}\frac{f''}{A^4} = \frac{2\kappa ^2}{A}(\phi ' + 3\mathcal {H}\phi - \mathcal {L}\psi )\,, \end{aligned}$$

with

84 $$\begin{aligned} \mathcal {E} = 4\mathcal {H}^2(\mathcal {H} + \mathcal {L}) - 2(2\mathcal {H} + \mathcal {L})\mathcal {H}' - \mathcal {L}'' = -A^2\frac{G'}{3}, \end{aligned}$$

and

85 $$\begin{aligned} G = \frac{6\mathcal {H}(\mathcal {H} + \mathcal {L}) + 3\mathcal {L}'}{A^2}, \end{aligned}$$

Note that the last three branches of cosmological dynamics allow for a particular classification of their solutions in case the right hand side of the connection field equations vanishes, e.g., for vanishing hypermomentum. Further assuming $u^2 < 0$, i.e., negative spatial curvature, the connection field equations (76) are solved by $\mathcal {L} = \pm iu$. Choosing the lower sign, the metric field equations (75) then reduce to

86a $$\begin{aligned}&12\mathcal {H}(\mathcal {H} + iu)\frac{f'}{A^2} - f = 2\kappa ^2\rho \,, \end{aligned}$$

86b $$\begin{aligned}&-48(\mathcal {H} + iu)^2(\mathcal {H}' - \mathcal {H}^2 - iu\mathcal {H})\frac{f''}{A^4}\nonumber \\&\quad - 4(\mathcal {H}' + 2\mathcal {H}^2 + 3iu\mathcal {H} - u^2)\frac{f'}{A^2} + f = 2\kappa ^2p\,, \end{aligned}$$

while for the upper sign one obtains an equivalent set of equations up to the trivial redefinition $u \mapsto -u$. It follows that in these cases the cosmological dynamics in *f*(*G*) gravity become identical to the vector branch of the metric teleparallel *f*(*T*) class of theories [15, 19]. Since in this case the connection degree of freedom $\mathcal {L}$ is fixed by an algebraic equation we do not obtain a dynamical contribution of it to the metric evolution. So, even if the resulting equations would give rise to a dark energy like effect, i.e. accelerated expansion at late times, it should not be called dynamical as it would rather come from the choice of *f* and not a new degree of freedom. Similarly, setting $\mathcal {L} = 0$ solves the connection equations in (80) and (83) (again assuming that their right hand side vanishes), while in both cases the metric field equations reduce to (73) again, and thus reproduce the flat limiting case of *f*(*T*) cosmology. Any remaining solutions, given by $\mathcal {L} \ne \pm iu$ in the spatially curved case and $\mathcal {L} \ne 0$ in the spatially flat cases – except in 1a and 2a where the connection equations of motion are trivial – must then satisfy $\mathcal {E} = 0$, leading to $G=G_0$ where $G_0$ is a constant. This follows directly from the form of the connection equations of motion, where all terms on the left side are proportional to $f''\partial _\rho G$. In general the left side is then an algebraic combination of derivatives of *G*, but since in our cosmological setup *G* depends only time all terms are proportional to $G'$. Since *G* contains first derivatives of $\mathcal {L}$ the equation $G=G_0$ can be seen as a first order differential equation for $\mathcal {L}$. For example, for the case 1b one finds

87 $$\begin{aligned}&\mathcal {L}=\frac{\mathcal {L}_0}{A^2}{+}\frac{1}{3A^2}\int \textrm{d}\eta \, A^2(G_0A^2{-}6\mathcal {H}^2), \end{aligned}$$

with $\mathcal {L}_0$ an integration constant and $\eta$ conformal time. However, one can easily see that for constant $G=G_0$ the metric equations of motion become88which are nothing but the GR Friedmann equations with rescaled $\kappa ^2$ and an effective cosmological constant. This case does also not lead to a dynamical dark energy, since only *f* and its derivatives evaluated at $G_0$ enter the metric equations, which are fixed and do not evolve, though one may see $G_0$ as another integration constant of solving $\mathcal {E}=0$ for $\mathcal {L}$. In the presence of hypermomentum the connection equations become more involved and will generally lead to $G'\ne 0$ and more interesting solutions, but it is curious to see that in its absence *f*(*G*) gravity does not involve a dynamical connection. Either $\mathcal {L}$ is fixed by an algebraic equation or it leads to constant *G*.

### General teleparallel quadratic gravity

The next class of teleparallel gravity theories we study is known as general teleparallel quadratic gravity [6]. Its action is an arbitrary linear combination of the 11 scalars which are quadratic in torsion and nonmetricity, and hence takes the form89with 11 constant coefficients either parametrized as $a_{1,\ldots ,3}, b_{1,\ldots ,3}, c_{1,\ldots ,5}$ or $k_{1\,\ldots ,11}$. While the former parametrization gives more insight into the split between torsion and nonmetricity, and will be more convenient for the purposes of this article, the latter turns out to be more convenient to express the field equations, which can be written in the form [10]90and

91 $$\begin{aligned}{} & {} \nabla _{\tau }Z_{\mu }{}^{\nu \tau } - M^{\omega }{}_{\tau \omega }Z_{\mu }{}^{\nu \tau } \nonumber \\{} & {} \quad = 2\kappa ^2(\nabla _{\tau }H_{\mu }{}^{\nu \tau } - M^{\omega }{}_{\tau \omega }H_{\mu }{}^{\nu \tau }), \end{aligned}$$

where we introduced the abbreviations

92a $$\begin{aligned} U^{\mu \nu }= & {} k_1(M^{\mu \rho \sigma }M^{\nu }{}_{\rho \sigma } - M^{\rho \mu }{}_{\sigma }M_{\rho }{}^{\nu \sigma } \nonumber \\{} & {} - M_{\rho \sigma }{}^{\mu }M^{\rho \sigma \nu }) - k_2M_{\rho }{}^{\sigma (\mu }M\nonumber \\{} & {} + k_3(M^{\mu \rho \sigma }M^{\nu }{}_{\sigma \rho } - 2M^{\rho \sigma (\mu }M_{\rho }{}^{\nu )}{}_\sigma )\nonumber \\{} & {} - k_4M^{\rho \mu }{}_{\sigma }M^{\sigma \nu }{}_{\rho } - k_5M^{\rho \sigma \mu }M_{\sigma \rho }{}^{\nu } \nonumber \\{} & {} + k_6M^{\mu \rho }{}_{\rho }M^{\nu \sigma }{}_{\sigma } - k_7M^{\rho \mu }{}_{\rho }M^{\sigma \nu }{}_{\sigma }\nonumber \\{} & {} - k_8M_{\rho }{}^{\rho \mu }M_{\sigma }{}^{\sigma \nu } - k_9M_{\rho }{}^{\rho (\mu }M_{\sigma }{}^{\nu )\sigma } \nonumber \\{} & {} - (2k_6M_{\rho \sigma }{}^{\sigma } + k_{11}M_{\sigma \rho }{}^{\sigma } + k_{10}M^{\sigma }{}_{\sigma \rho })M^{\rho (\mu \nu )},\nonumber \\ \end{aligned}$$

as well as

92b $$\begin{aligned} V^{\rho \mu \nu }= & {} -2k_6g^{\rho (\mu }M^{\nu )\sigma }{}_{\sigma } - k_{11}g^{\rho (\mu }M_{\sigma }{}^{\nu )\sigma } \nonumber \\{} & {} - k_{10}M_{\sigma }{}^{\sigma (\mu }g^{\nu )\rho } + (k_4 - k_5 - k_1 - k_3)M^{(\mu \nu )\rho } \nonumber \\{} & {} + (k_5 - k_4 - k_1 - k_3)M^{(\mu |\rho |\nu )}\nonumber \\{} & {} + (k_1 - k_2 + k_3 - k_4 - k_5)M^{\rho (\mu \nu )}+\frac{1}{2}g^{\mu \nu } \nonumber \\{} & {} \times \Bigg [(2k_6 - k_{10} - k_{11})M^{\rho \sigma }{}_{\sigma }+ (k_{11} - 2k_7 - k_9) \nonumber \\{} & {} M^{\sigma \rho }{}_{\sigma } + (k_{10} - 2k_8 - k_9)M_{\sigma }{}^{\sigma \rho }\Bigg ]\nonumber \\ \end{aligned}$$

and

92c $$\begin{aligned} Z_{\mu }{}^{\nu \rho }= & {} 2k_1M_{\mu }{}^{\nu \rho } + k_2(M^{\nu \rho }{}_{\mu } + M^{\rho }{}_{\mu }{}^{\nu }) \nonumber \\{} & {} + 2k_3M_{\mu }{}^{\rho \nu } + 2k_4M^{\rho \nu }{}_{\mu } + 2k_5M^{\nu }{}_{\mu }{}^{\rho } \nonumber \\{} & {} + 2k_6M_{\mu \sigma }{}^{\sigma }g^{\nu \rho } + 2k_7M^{\sigma \nu }{}_{\sigma }\delta _{\mu }^{\rho }\nonumber \\{} & {} + 2k_8M_{\sigma }{}^{\sigma \rho }\delta _{\mu }^{\nu } + k_9(M_{\sigma }{}^{\rho \sigma }\delta _{\mu }^{\nu } + M_{\sigma }{}^{\sigma \nu }\delta _{\mu }^{\rho }) \nonumber \\{} & {} +k_{10}(M^{\rho \sigma }{}_{\sigma }\delta _{\mu }^{\nu }+ M^{\sigma }{}_{\sigma \mu }g^{\nu \rho }) \nonumber \\{} & {} + k_{11}(M^{\nu \sigma }{}_{\sigma }\delta _{\mu }^{\rho } + M^{\sigma }{}_{\mu \sigma }g^{\nu \rho }). \end{aligned}$$

The two parametrizations are related by

93 $$\begin{aligned} k_1= & {} 2a_1 - b_1 + 2c_1, \quad k_2 = -2a_2 + b_1 + 2c_2, \nonumber \\ k_9= & {} -2a_3 + 2b_2 - b_3 + 2c_5, \quad k_4 = a_2 + c_2, \nonumber \\ k_5= & {} a_2 - b_1 + 2c_1, k_6 = c_4, \quad k_7 = a_3 + b_3 + c_4, \nonumber \\ k_8= & {} a_3 - 2b_2 + 4c_3, \quad k_3 = -2a_1 + b_1 + c_2, \nonumber \\ k_{10}= & {} -b_3 + 2c_5, \quad k_{11} = b_3 + 2c_4. \end{aligned}$$

Note that for the values

94 $$\begin{aligned} a_1= & {} \frac{1}{4}, \quad a_2 = \frac{1}{2}, \quad a_3 = -1, \quad b_1 = 1, \quad b_2 = -1, \nonumber \\ b_3= & {} 1, \quad c_1 = \frac{1}{4}, \quad c_2 = -\frac{1}{2}, \quad c_3 = -\frac{1}{4}, \quad c_4 = 0, \quad \nonumber \\ c_5= & {} \frac{1}{2}, \end{aligned}$$

or equivalently,

95 $$\begin{aligned} k_{11}= & {} -k_2 = 1, \nonumber \\ k_1= & {} k_3 = k_4 = k_5 = k_6 = k_7 = k_8 = k_9 = k_{10} = 0, \end{aligned}$$

we obtain the GTEGR action (15). We now study the cosmological dynamics of this class of theories. While deriving the cosmological field equations, one finds that not all 11 coefficients enter these equations independently, but only a limited number of linear combinations appears. A suitable parametrization of these linear combinations is given by

96 $$\begin{aligned} z_1= & {} -\frac{2a_1 + a_2 + 3a_3}{2}, \quad z_2 = \frac{3}{2}(3a_2 + a_3 - 2a_1), \nonumber \\ z_4= & {} 2a_1 + a_2 + 3a_3 - 2b_1 - 6b_2 + 4c_1 + 12c_3, \nonumber \\ z_7= & {} 2c_3 + c_5, \quad z_3 = c_2 - c_4 + c_5, \quad z_5 = b_1 + 3b_2 - 4c_1\nonumber \\ {}{} & {} - 12c_3, \nonumber \\ z_6= & {} b_2 + b_3 - 4c_3 - 2c_5, \quad z_8 = c_1 + c_2 + c_3 + c_4 + c_5.\nonumber \\ \end{aligned}$$

This particular parametrization is chosen here as it will turn out to simplify the cosmological field equations as much as possible, and that for GTEGR it is simply given by

97 $$\begin{aligned} z_1 = 1, \quad z_2 = z_3 = z_4 = z_5 = z_6 = z_7 = z_8 = 0. \end{aligned}$$

Hence, we can easily see which new terms arise from modifications of GTEGR and how they alter the dynamics. We now display the cosmological field equations, and give a few remarks on their general properties. We start with the branch 2a, for which they read

98a $$\begin{aligned}&6(z_1 + z_2)u^2 + 6(z_1 - 2z_6 - 4z_7)\mathcal {H}^2 - 6(z_6 + 2z_7)\mathcal {H}' \nonumber \\&\quad - 6z_6\mathcal {K}' - 8z_8\mathcal {L}' - 4z_8\mathcal {L}^2 - 3z_4\mathcal {K}^2 \nonumber \\ {}&\quad - 6(z_4 + z_5 + 2z_6)\mathcal {H}\mathcal {K} \nonumber \\&\quad - 2(3z_6 + 6z_7 + 8z_8)\mathcal {H}\mathcal {L} - 6z_6\mathcal {K}\mathcal {L} = 2\kappa ^2A^2\rho \,, \end{aligned}$$

98b $$\begin{aligned}&\quad -2(z_1 + z_2)u^2 - 2(z_1 + 2z_4 + 2z_5)\mathcal {H}^2 \nonumber \\&\quad - 2(2z_1 + z_4 + z_5)\mathcal {H}' + 2z_5\mathcal {K}' + 4z_7\mathcal {L}' \nonumber \\&\quad - 3z_4\mathcal {K}^2 - 4z_8\mathcal {L}^2 - 2(3z_4 + z_5)\mathcal {H}\mathcal {K}\nonumber \\&\quad - 2(3z_6 + 2z_7)\mathcal {H}\mathcal {L} - 6z_6\mathcal {K}\mathcal {L} = 2\kappa ^2A^2p\,, \end{aligned}$$

98c $$\begin{aligned}&\quad -6(z_6 + 2z_7)(2\mathcal {H}^2 + \mathcal {H}') - 6z_6\mathcal {K}' - 8z_8\mathcal {L}'\nonumber \\&\quad - 4\mathcal {H}(3z_6\mathcal {K} + 4z_8\mathcal {L}) = 2\kappa ^2A(\omega ' + 3\mathcal {H}\omega )\,, \end{aligned}$$

98d $$\begin{aligned}&\quad -2(z_4 + z_5)(2\mathcal {H}^2 + \mathcal {H}') - 2z_4\mathcal {K}' - 2z_6\mathcal {L}' - 4\mathcal {H}(z_4\mathcal {K} \nonumber \\&\quad + z_6\mathcal {L}) = 2\kappa ^2A(\phi ' + 3\mathcal {H}\phi )\,. \end{aligned}$$

Note that both $\mathcal {K}$ and $\mathcal {L}$ appear as dynamical quantities with first-order time derivatives, and that the dynamics does not depend on the parameter $z_3$ obtained from the action parameters. The same holds true for the branch 1a, which is obtained taking the limit $u \rightarrow 0$, and leads to the field equations

99a $$\begin{aligned}&6(z_1 - 2z_6 - 4z_7)\mathcal {H}^2 - 6(z_6 + 2z_7)\mathcal {H}' - 6z_6\mathcal {K}' - 8z_8\mathcal {L}' \nonumber \\&\quad - 4z_8\mathcal {L}^2 - 3z_4\mathcal {K}^2 - 6(z_4 + z_5 + 2z_6)\mathcal {H}\mathcal {K} \nonumber \\&\quad - 2(3z_6 + 6z_7 + 8z_8)\mathcal {H}\mathcal {L} - 6z_6\mathcal {K}\mathcal {L} = 2\kappa ^2A^2\rho \,, \end{aligned}$$

99b $$\begin{aligned}&\quad -2(z_1 + 2z_4 + 2z_5)\mathcal {H}^2 - 2(2z_1 + z_4 + z_5)\mathcal {H}' \nonumber \\&\quad + 2z_5\mathcal {K}' + 4z_7\mathcal {L}'- 3z_4\mathcal {K}^2 - 4z_8\mathcal {L}^2 - 2(3z_4 + z_5)\mathcal {H}\mathcal {K} \nonumber \\&\quad - 2(3z_6 + 2z_7)\mathcal {H}\mathcal {L} - 6z_6\mathcal {K}\mathcal {L} = 2\kappa ^2A^2p\,, \end{aligned}$$

99c $$\begin{aligned}&\quad -6(z_6 + 2z_7)(2\mathcal {H}^2 + \mathcal {H}') - 6z_6\mathcal {K}' - 8z_8\mathcal {L}'\nonumber \\&\quad - 4\mathcal {H}(3z_6\mathcal {K} + 4z_8\mathcal {L}) = 2\kappa ^2A(\omega ' + 3\mathcal {H}\omega )\,, \end{aligned}$$

99d $$\begin{aligned}&\quad -2(z_4 + z_5)(2\mathcal {H}^2 + \mathcal {H}') - 2z_4\mathcal {K}' - 2z_6\mathcal {L}' - 4\mathcal {H}(z_4\mathcal {K} \nonumber \\&\quad + z_6\mathcal {L}) = 2\kappa ^2A(\phi ' + 3\mathcal {H}\phi )\,. \end{aligned}$$

Note that one can also obtain the cosmological dynamics of branch 1a from 2a by setting $z_2=-z_1$, which only affects the curvature term $(z_1+z_2)u^2$ as $z_2$ only appears here. Hence one can obtain the same dynamics as in spatially flat cosmological solutions also in curved spaces by setting $z_1+z_2=0$, regardless of their spatial curvature. We then continue with the branch 2b, for which the cosmological field equations become significantly more involved, and consist of the metric field equations

100a $$\begin{aligned}&3(2z_1 - 2z_3 + z_4 + z_5 + 3z_6 + 4z_7 + 4z_8)u^2 \nonumber \\&\quad - 3(4z_3 - 2z_4 - 3z_5 - 7z_6 - 18z_7 - 4z_8)\mathcal {H}\mathcal {L} \nonumber \\&\quad - 9(z_3 + z_7 - 2z_8)\frac{u^4}{\mathcal {L}^2} + 3(z_5 - 3z_6 - 2z_7 + 4z_8)u^2\frac{\mathcal {K}}{\mathcal {L}}\nonumber \\&\quad -3(4z_3 - z_5 + 3z_6 + 2z_7 - 4z_8)u^2\frac{\mathcal {H}}{\mathcal {L}} \nonumber \\&\quad + 6(z_1 - 2z_6 - 4z_7)\mathcal {H}^2 - 6(z_6 + 2z_7)\mathcal {H}'\nonumber \\&\quad - 2(3z_4 + 3z_5 + 3z_6 - 6z_7 - 8z_8)\mathcal {H}\mathcal {K} \nonumber \\&\quad + 2(3z_6 + 6z_7 + 8z_8)\frac{\mathcal {H}\mathcal {L}'}{\mathcal {L}}- (3z_4 - 6z_6 + 4z_8)\mathcal {K}^2 \nonumber \\&\quad + 3(z_3 - z_4 - z_5 - 3z_6 \nonumber \\&\quad - 5z_7 - 2z_8) \mathcal {L}^2 + 4z_8\frac{2\mathcal {L}\mathcal {L}'' - 3\mathcal {L}'^2}{\mathcal {L}^2} + (6z_6 - 8z_8)\nonumber \\&\quad \times \frac{\mathcal {K}\mathcal {L}' - \mathcal {L}\mathcal {K}'}{\mathcal {L}} + 3(2z_4 + z_5 + z_6 - 2z_7 - 4z_8)\mathcal {K}\mathcal {L} \nonumber \\&= 2\kappa ^2A^2\rho \,, \end{aligned}$$

100b $$\begin{aligned}&3(z_3 - z_4 - z_5 - 3z_6 - 5z_7 - 2z_8)\mathcal {L}^2 \nonumber \\&\quad - (4z_3 + z_5 - 3z_6 - 2z_7 + 4z_8)u^2\frac{\mathcal {H}}{\mathcal {L}} \nonumber \\&\quad - (2z_1 - 2z_3 + z_4 + z_5 + 3z_6 + 4z_7 + 4z_8)u^2\nonumber \\&\quad + 2(2z_3 - z_5 - 3z_6 - 10z_7 - 4z_8)\mathcal {L}' \nonumber \\&\quad - (4z_3 - 6z_4 - 5z_5 - 9z_6 - 14z_7 + 4z_8)\mathcal {H}\mathcal {L} \nonumber \\&\quad + 3(2z_4 + z_5 + z_6 - 2z_7 - 4z_8)\mathcal {K}\mathcal {L} \nonumber \\&\quad + 2(z_5 - 2z_7)\mathcal {K}' - 2(2z_1 + z_4 + z_5)\mathcal {H}' \nonumber \\&\quad - 2(z_1 + 2z_4 + 2z_5) \mathcal {H}^2 - (z_3 + z_7 - 2z_8) \frac{u^4}{\mathcal {L}^2} \nonumber \\&\quad - (z_5 - 3z_6 - 2z_7 + 4z_8)u^2\frac{\mathcal {K}}{\mathcal {L}} + 4z_3u^2\frac{\mathcal {L}'}{\mathcal {L}^2} \nonumber \\&\quad + 2(3z_6 + 2z_7)\frac{\mathcal {H}\mathcal {L}'}{\mathcal {L}} + 2(3z_6 - 4z_8)\frac{\mathcal {K}\mathcal {L}'}{\mathcal {L}} \nonumber \\&\quad - 2(3z_4 + z_5 - 3z_6 - 2z_7)\mathcal {H}\mathcal {K} - (3z_4 - 6z_6 + 4z_8)\mathcal {K}^2\nonumber \\&\quad + 4(z_7 - z_8)\frac{\mathcal {L}'^2}{\mathcal {L}^2} - 4z_7\frac{\mathcal {L}''}{\mathcal {L}} = 2\kappa ^2A^2p\,, \end{aligned}$$

as well as the connection field equations

100c $$\begin{aligned}&4(3z_6 - 4z_8)\mathcal {H}\mathcal {K} + 6(z_3 + z_7 - 2z_8)\frac{u^4}{\mathcal {L}^2} \nonumber \\&\quad + 3(4z_3 - z_5 + 3z_6 + 2z_7 - 4z_8)u^2\frac{\mathcal {H}}{\mathcal {L}} \nonumber \\&\quad + 6(z_6 + 2z_7)(2\mathcal {H}^2 + \mathcal {H}') \nonumber \\&\quad + 3(4z_3 - 2z_4 - 3z_5 - 7z_6 - 18z_7 - 4z_8)\mathcal {H}\mathcal {L} \nonumber \\&\quad - 6(z_3 - z_4 - z_5 - 3z_6 - 5z_7 - 2z_8)\mathcal {L}^2 \nonumber \\&\quad - 3(z_5 - 3z_6 - 2z_7 + 4z_8)u^2\frac{\mathcal {K}}{\mathcal {L}} \nonumber \\&\quad - 3(2z_4 + z_5 + z_6 - 2z_7 - 4z_8)\mathcal {K}\mathcal {L} + (6z_6 - 8z_8)\mathcal {K}' \nonumber \\&\quad + 8z_8\frac{\mathcal {L}'^2 - \mathcal {L}\mathcal {L}'' - 2\mathcal {H}\mathcal {L}\mathcal {L}'}{\mathcal {L}^2}\nonumber \\&\quad = 2\kappa ^2A\left( \omega ' + 3\mathcal {H}\omega + 3\mathcal {L}\chi - 3\frac{u^2}{\mathcal {L}}\psi \right) \,, \end{aligned}$$

100d $$\begin{aligned}&2(z_4 + z_5)(2\mathcal {H}^2 + \mathcal {H}') + 2(z_3 + z_7 - 2z_8) \frac{u^4}{\mathcal {L}^2} \nonumber \\&\quad - 2(z_3 - z_4 - z_5 - 3z_6 - 5z_7 - 2z_8)\mathcal {L}^2 \nonumber \\&\quad - (z_5 - 3z_6 - 2z_7 + 4z_8)u^2\frac{\mathcal {L}'}{\mathcal {L}^2} \nonumber \\&\quad + (4z_3 - 6z_4 - 5z_5 - 9z_6 - 14z_7 + 4z_8)\mathcal {H}\mathcal {L} \nonumber \\&\quad - (2z_4 + z_5 + z_6 - 2z_7 - 4z_8)(\mathcal {K}\mathcal {L} + \mathcal {L}') \nonumber \\&\quad + (4z_3 + z_5 - 3z_6 - 2z_7 + 4z_8)u^2\frac{\mathcal {H}}{\mathcal {L}} \nonumber \\&\quad + 2(z_4 - z_6)(2\mathcal {H}\mathcal {K} + \mathcal {K}') - (z_5 - 3z_6 - 2z_7 + 4z_8)u^2\frac{\mathcal {K}}{\mathcal {L}} \nonumber \\&\quad + 2z_6\frac{\mathcal {L}'^2 - \mathcal {L}\mathcal {L}'' - 2\mathcal {H}\mathcal {L}\mathcal {L}'}{\mathcal {L}^2} \nonumber \\&\quad = 2\kappa ^2A\left( \phi ' + 3\mathcal {H}\phi + \mathcal {L}\chi - \frac{u^2}{\mathcal {L}}\psi \right) \,. \end{aligned}$$

We see that in contrast to the two previously studied branches, $\mathcal {L}$ now also appears with second-order time derivatives and the parameter $z_3$ enters the equations. This holds true also for the remaining flat branches. For the branch 1c we find the dynamical equations

101a $$\begin{aligned}&2(3z_6 + 6z_7 + 8z_8)\frac{\mathcal {H}\mathcal {L}'}{\mathcal {L}} \nonumber \\&\quad - 3(4z_3 - 2z_4 - 3z_5 - 7z_6 - 18z_7 - 4z_8)\mathcal {H}\mathcal {L} \nonumber \\&\quad + 6(z_1 - 2z_6 - 4z_7)\mathcal {H}^2 - 6(z_6 + 2z_7)\mathcal {H}'- 2(3z_4 + 3z_5 \nonumber \\&\quad + 3z_6 - 6z_7 - 8z_8)\mathcal {H}\mathcal {K}- (3z_4 - 6z_6 + 4z_8) \nonumber \\&\quad \times \mathcal {K}^2 + 3(z_3 - z_4 - z_5 - 3z_6 - 5z_7 - 2z_8)\nonumber \\&\quad \times \mathcal {L}^2 + 4z_8\frac{2\mathcal {L}\mathcal {L}'' - 3\mathcal {L}'^2}{\mathcal {L}^2} + (6z_6 - 8z_8)\frac{\mathcal {K}\mathcal {L}' - \mathcal {L}\mathcal {K}'}{\mathcal {L}} \nonumber \\&\quad + 3(2z_4 + z_5 + z_6 - 2z_7 - 4z_8)\mathcal {K}\mathcal {L} = 2\kappa ^2A^2\rho \,, \end{aligned}$$

101b $$\begin{aligned}&3(z_3 - z_4 - z_5 - 3z_6 - 5z_7 - 2z_8)\mathcal {L}^2 \nonumber \\&\quad - (4z_3 - 6z_4 - 5z_5 - 9z_6 - 14z_7 + 4z_8)\mathcal {H}\mathcal {L} \nonumber \\&\quad + 2(2z_3 - z_5 - 3z_6 - 10z_7 - 4z_8)\mathcal {L}' \nonumber \\&\quad + 3(2z_4 + z_5 + z_6 - 2z_7 - 4z_8) \mathcal {K}\mathcal {L} - 4z_7\frac{\mathcal {L}''}{\mathcal {L}} \nonumber \\&\quad + 2(3z_6 + 2z_7)\frac{\mathcal {H}\mathcal {L}'}{\mathcal {L}} - 2(2z_1 + z_4 + z_5)\mathcal {H}' \nonumber \\&\quad - 2(z_1 + 2z_4 + 2z_5)\mathcal {H}^2+ 2(3z_6 - 4z_8)\frac{\mathcal {K}\mathcal {L}'}{\mathcal {L}} \nonumber \\&\quad + 2(z_5 - 2z_7)\mathcal {K}' - 2(3z_4 + z_5 - 3z_6 - 2z_7) \mathcal {H}\mathcal {K} \nonumber \\&\quad - (3z_4 - 6z_6 + 4z_8)\mathcal {K}^2 + 4(z_7 - z_8) \frac{\mathcal {L}'^2}{\mathcal {L}^2} = 2\kappa ^2A^2p\,, \end{aligned}$$

101c $$\begin{aligned}&4(3z_6 - 4z_8)\mathcal {H}\mathcal {K} - 6(z_3 - z_4 - z_5 - 3z_6 - 5z_7 - 2z_8) \mathcal {L}^2 \nonumber \\&\quad + 6(z_6 + 2z_7)(2\mathcal {H}^2 + \mathcal {H}')\nonumber \\&\quad + 3(4z_3 - 2z_4 - 3z_5 - 7z_6 - 18z_7 - 4z_8)\mathcal {H}\mathcal {L}&\nonumber \\&\quad - 3(2z_4 + z_5 + z_6 - 2z_7 - 4z_8)\mathcal {K}\mathcal {L} + (6z_6 - 8z_8)\mathcal {K}' \nonumber \\&\quad + 8z_8\frac{\mathcal {L}'^2 - \mathcal {L}\mathcal {L}''- 2\mathcal {H}\mathcal {L}\mathcal {L}'}{\mathcal {L}^2} = 2\kappa ^2A\left( \omega ' + 3\mathcal {H}\omega + 3\mathcal {L}\chi \right) \,, \end{aligned}$$

101d $$\begin{aligned}&2(z_4 {+} z_5)(2\mathcal {H}^2 {+} \mathcal {H}') {-} 2(z_3 {-} z_4 {-} z_5 {-} 3z_6 {-} 5z_7 {-} 2z_8)\mathcal {L}^2 \nonumber \\&\quad + 2(z_4 - z_6)(2\mathcal {H}\mathcal {K}+ \mathcal {K}')+ (4z_3 - 6z_4 - 5z_5 \nonumber \\&\quad - 9z_6 - 14z_7 + 4z_8) \mathcal {H}\mathcal {L}- (2z_4 + z_5 + z_6 \nonumber \\&\quad - 2z_7 - 4z_8)(\mathcal {K}\mathcal {L} + \mathcal {L}')\nonumber \\&\quad + 2z_6\frac{\mathcal {L}'^2 - \mathcal {L}\mathcal {L}'' - 2\mathcal {H}\mathcal {L}\mathcal {L}'}{\mathcal {L}^2} = 2\kappa ^2A\left( \phi ' + 3\mathcal {H}\phi + \mathcal {L}\chi \right) \,, \end{aligned}$$

while in the branch 1b they become

102a $$\begin{aligned}&-2(3z_6 + 6z_7 + 8z_8)\frac{\mathcal {H}\mathcal {L}'}{\mathcal {L}}- 3(4z_3 - z_5 + 3z_6 \nonumber \\&\quad + 2z_7 - 4z_8)\mathcal {H}\mathcal {L}+ 6(z_1 - 2z_6 - 4z_7)\mathcal {H}^2 \nonumber \\&\quad - 2(3z_4 + 3z_5 + 3z_6 - 6z_7 - 8z_8) \mathcal {H}\mathcal {K} \nonumber \\&\quad - 6(z_6 + 2z_7)\mathcal {H}' - (3z_4 - 6z_6 + 4z_8)\mathcal {K}^2\nonumber \\&\quad - 9(z_3 + z_7 - 2z_8)\mathcal {L}^2 - 4z_8\frac{2\mathcal {L}\mathcal {L}'' - \mathcal {L}'^2}{\mathcal {L}^2} \nonumber \\&\quad - (6z_6 - 8z_8)\frac{\mathcal {K}\mathcal {L}' + \mathcal {L}\mathcal {K}'}{\mathcal {L}}+ 3(z_5 - 3z_6 - 2z_7 + 4z_8)\nonumber \\&\quad \mathcal {K}\mathcal {L} = 2\kappa ^2A^2\rho \,, \end{aligned}$$

102b $$\begin{aligned}&2(z_5 - 2z_7)\mathcal {K}' - (z_3 + z_7 - 2z_8)\mathcal {L}^2 \nonumber \\&\quad - (4z_3 + z_5 - 3z_6 - 2z_7 + 4z_8)\mathcal {H}\mathcal {L} \nonumber \\&\quad - 4z_3\mathcal {L}' - (z_5 - 3z_6 - 2z_7 + 4z_8)\mathcal {K}\mathcal {L} \nonumber \\&\quad + 4z_7\frac{\mathcal {L}''}{\mathcal {L}} - (3z_4 - 6z_6 + 4z_8)\mathcal {K}^2 \nonumber \\&\quad - 2(2z_1 + z_4 + z_5)\mathcal {H}' - 2(z_1 + 2z_4 + 2z_5)\mathcal {H}^2 \nonumber \\&\quad - 2(3z_6 - 4z_8)\frac{\mathcal {K}\mathcal {L}'}{\mathcal {L}}- 2(3z_6 + 2z_7)\frac{\mathcal {H}\mathcal {L}'}{\mathcal {L}}s \nonumber \\&\quad - 2(3z_4 + z_5 - 3z_6 - 2z_7)\mathcal {H}\mathcal {K} \nonumber \\&\quad - 4(z_7 + z_8)\frac{\mathcal {L}'^2}{\mathcal {L}^2} = 2\kappa ^2A^2p\,, \end{aligned}$$

102c $$\begin{aligned}&4(3z_6 - 4z_8)\mathcal {H}\mathcal {K}+ 6(z_3 + z_7 - 2z_8)\mathcal {L}^2\nonumber \\&\quad + (6z_6 - 8z_8)\mathcal {K}+ 6(z_6 + 2z_7)(2\mathcal {H}^2 + \mathcal {H}') '\nonumber \\&\quad + 3(4z_3 - z_5 + 3z_6 + 2z_7 - 4z_8)\mathcal {H}\mathcal {L} \nonumber \\&\quad - 3(z_5 - 3z_6 - 2z_7 + 4z_8)\mathcal {K}\mathcal {L} '\nonumber \\&\quad - 8z_8\frac{\mathcal {L}'^2 - \mathcal {L}\mathcal {L}'' - 2\mathcal {H}\mathcal {L}\mathcal {L}'}{\nonumber }\\ {\mathcal {L}^2}&= 2\kappa ^2A\left( \omega ' + 3\mathcal {H}\omega - 3\mathcal {L}\psi \right) \,, \end{aligned}$$

102d $$\begin{aligned}&2(z_4 + z_5)(2\mathcal {H}^{2} + \mathcal {H}') + 2(z_3 + z_7 - 2z_8)\mathcal {L}^{2}\nonumber \\&\quad - 2z_6\frac{\mathcal {L}'^{2} - \mathcal {L}\mathcal {L}'' - 2\mathcal {H}\mathcal {L}\mathcal {L}'}{\mathcal {L}^{2}} \nonumber \\&\quad + 2(z_4 - z_6)(2\mathcal {H}\mathcal {K} + \mathcal {K}')'\nonumber \\&\quad + (4z_3 + z_5 - 3z_6 - 2z_7 + 4z_8)\mathcal {H}\mathcal {L} \nonumber \\&\quad + (z_5 - 3z_6 - 2z_7 + 4z_8)(\mathcal {L}' -\mathcal {K}\mathcal {L})'\nonumber \\&\quad = 2\kappa ^{2}A\left( \phi ' + 3\mathcal {H}\phi - \mathcal {L}\psi \right) \,. \end{aligned}$$

Studying the full dynamics arising for the different branches and all possible parameter choices is a major task that would exceed the scope of this article. We therefore restrict ourselves to a few particular classes of theories which are motivated by a field theoretical perspective, which considers general teleparallel quadratic gravity as a theory of three dynamical fields $h_{\mu \nu }, H_{\mu \nu }, B_{\mu \nu }$ propagating on Minkowski spacetime, where $h_{\mu \nu }$ is the metric perturbation, while $H_{\mu \nu }$ and $B_{\mu \nu }$ are the symmetric and antisymmetric parts of the connection perturbation [6]. The first class of theories we mention here are those from which the field $B_{\mu \nu }$, which is a two-form which transforms non-trivially under local Lorentz transformations of the teleparallel connection, is absent. From [6], such kind of local Lorentz invariance is realized by the conditions

103 $$\begin{aligned} a_2 - 2a_1 = a_3 + 4a_1 = b_3 - b_1 = 0. \end{aligned}$$

Theories satisfying these conditions necessarily have $z_2 = 0$. Observe that $z_2$ only enters the field equations (98) in the branch 2a, where it governs the terms involving the curvature parameter $u^2$, and is absent in all other branches. This is related to the fact that $B_{\mu \nu }$ contributes to the axial torsion component, which is non-vanishing only in the branch 2a, and enforced to vanish by the cosmological symmetry in the other branches. Alternatively, one may consider the weaker condition that $B_{\mu \nu }$ is a propagating two-form with a $\textrm{U}(1)$ gauge symmetry. This is the case if the theory parameters satisfy the conditions

104 $$\begin{aligned} 2a_1 + a_2 + a_3 = b_3 - b_1 = 0, \end{aligned}$$

which by themselves have no immediate consequence on the linear combinations $z_{1,\ldots ,8}$ relevant in the cosmological dynamics, but will contribute if they are combined with other conditions as we will see below.

Another type of conditions on the parameters can be obtained that the two fields $h_{\mu \nu }$ and $H_{\mu \nu }$ propagate two massless spin-2 fields. This can be obtained if one enhances the diffeomorphism invariance of the theory by another gauge symmetry, such that $h_{\mu \nu }$ and $H_{\mu \nu }$ become individually invariant under diffeomorphisms. This is the case for

105 $$\begin{aligned} 2c_1 - c_5 = c_1 + c_3 = 2c_1 + c_2 + c_4 = b_1 + b_2 = 0, \end{aligned}$$

and implies $z_7 = z_8 = 0$. Further demanding that the two spin-2 fields decouple leads to the additional condition $b_1 = 4c_1$, which then further implies $z_5 = 0$. Finally, combining these conditions with the condition (104) of a propagating two-form yields another condition $z_6 = 0$. Under these conditions, the cosmological dynamics simplify significantly. For the branch 2a and its flat limit 1a, the variable $\mathcal {L}$ decouples completely, and only $\mathcal {K}$ contributes to the cosmological dynamics. For the remaining three branches, $\mathcal {L}$ remains in the cosmological field equations, but its equation of motion changes from a second-order to a first-order differential equation, as the second-order derivative terms disappear from the cosmological field equations.

The propagation of two massles spin-2 fields can also be achieved by imposing transverse diffeomorphisms and Weyl symmetry as an additional gauge symmetry. The former gives the condition $2c_1 + c_2 + c_4 = 0$, while the latter is realized by

106a $$\begin{aligned}&82(2a_1 + a_2)w_H - 2(c_2 + c_4 - 8c_3 - c_5)(w_h - w_H) \nonumber \\&\quad - b_1(2w_h - 3w_H) - b_2(4w_h - 7w_H) = 0\,, \end{aligned}$$

106b $$\begin{aligned}&b_1w_h + 2(b_2 + c_2 + c_4 + 2c_5)(w_h - w_H) - (2a_1 + a_2)\nonumber \\&\quad w_H = 0\,, \end{aligned}$$

106c $$\begin{aligned}&2(c_2 + c_4 - 8c_3 - c_5)(w_h - w_H) - (b_1 + 3b_2)w_H = 0\,, \end{aligned}$$

106d $$\begin{aligned}&2(c_2 + c_4 + 2c_5)(w_h - w_H) + b_1w_H = 0 \end{aligned}$$

with two constants $w_h$ and $w_H$. Imposing these conditions implies that the linear combinations appearing in the cosmological dynamics must satisfy the condition

107 $$\begin{aligned} (2z_1 + z_4 + 2z_5 - 2z_7)w_h = (z_5 - 2z_7)w_H. \end{aligned}$$

Imposing in addition the two-form gauge condition (104) yields a more involved set of equations among the cosmologically relevant parameters, and so we restrict ourselves to two special cases. For $w_H = 0$ the conditions become

108 $$\begin{aligned} 2z_1 + z_4 + 2z_5 - 2z_7 = z_5 + z_6 = 3z_7 + 2z_8 = 0, \end{aligned}$$

while in the case $w_h = w_H \ne 0$ one finds

109 $$\begin{aligned} z_1 = z_4 + z_5 = z_6 + 2z_7 = 0. \end{aligned}$$

The former set of conditions does not have any particular relevance for the cosmological dynamics. Under the latter conditions, however, the cosmological dynamics enjoy the curious property that all derivatives of the Hubble parameter $\mathcal {H}$ disappear from the equation, and it becomes a purely constrained quantity. In this case the dynamics is fully carried by the connection variables $\mathcal {K}$ and $\mathcal {L}$. The physical implications of this case, which still has 5 free parameters, as well as those of the other cases discussed above, need to be determined by a more detailed case-by-case analysis, which we defer to future work, and hence conclude our discussion of general teleparallel quadratic gravity at this point.

### Scalar-teleparallel gravity

As the last example, we consider a class of scalar-teleparallel gravity theories, which is defined by the action [10, 20]

110 $$\begin{aligned}&S_{\text {g}} = \frac{1}{2\kappa ^2}\textrm{d}^4x\sqrt{-g}\int \left[ -\mathcal {A}(\Phi )G - \mathcal {B}(\Phi )g^{\mu \nu }\partial _{\mu }\Phi \partial _{\nu }\Phi \right. \nonumber \\&\quad \left. + \mathcal {C}(\Phi )(2T_{\nu }{}^{\nu \mu } - Q^{\mu \nu }{}_{\nu } + Q_{\nu }{}^{\nu \mu })\partial _{\mu }\Phi - 2\kappa ^2\mathcal {V}(\Phi )\right] ,\nonumber \\ \end{aligned}$$

where $\Phi$ denotes a scalar field, and $\mathcal {A}, \mathcal {B}, \mathcal {C}, \mathcal {V}$ are free functions, whose choice determines a particular theory within this class. The field equations are given by the metric equation111the connection equation112and the scalar field equation113Note that in the case $\mathcal {C} = -\mathcal {A}'$ this class of theories reduces to the well-studied class of scalar-curvature theories [10, 20].

We then take a look at the cosmological dynamics. For the branch 2a the metric and scalar field equations become

114a $$\begin{aligned}&6\mathcal {A}(\mathcal {H}^2 + u^2) - \mathcal {B}\Phi '^2 - 6\mathcal {C}\mathcal {H}\Phi ' - 2\kappa ^2A^2\mathcal {V} = 2\kappa ^2A^2\rho ,\nonumber \\ \end{aligned}$$

114b $$\begin{aligned}&-2\mathcal {A}(2\mathcal {H}' + \mathcal {H}^2 + u^2) + (2\mathcal {C}' - \mathcal {B})\Phi '^2 - 2(2\mathcal {A}' + \mathcal {C})\mathcal {H}\Phi '\nonumber \\&\quad + 2\mathcal {C}\Phi '' + 2\kappa ^2A^2\mathcal {V} = 2\kappa ^2A^2p\,, \end{aligned}$$

114c $$\begin{aligned}&6\mathcal {C}\mathcal {H}' + 6(2\mathcal {C} + \mathcal {A}')\mathcal {H}^2 - 6\mathcal {A}'u^2 \nonumber \\&\quad + 2\mathcal {B}(\Phi '' + 2\mathcal {H}\Phi ') + \mathcal {B}'\Phi '^2 + 2\kappa ^2A^2\mathcal {V}' = 0\,. \end{aligned}$$

The left hand side of the connection field equations vanishes identically, and so these equations are accompanied by the hypermomentum conservation equations

115 $$\begin{aligned} \omega ' + 3\mathcal {H}\omega = \phi ' + 3\mathcal {H}\phi = 0. \end{aligned}$$

By taking the limit $u \rightarrow 0$, this branch reduces to the branch 1a, and the field equations become

116a $$\begin{aligned}&6\mathcal {A}\mathcal {H}^2 - \mathcal {B}\Phi '^2 - 6\mathcal {C}\mathcal {H}\Phi ' - 2\kappa ^2A^2\mathcal {V} = 2\kappa ^2A^2\rho \,, \end{aligned}$$

116b $$\begin{aligned}&-2\mathcal {A}(2\mathcal {H}' + \mathcal {H}^2) + (2\mathcal {C}' - \mathcal {B})\Phi '^2\nonumber \\&\quad - 2(2\mathcal {A}' + \mathcal {C})\mathcal {H}\Phi ' + 2\mathcal {C}\Phi ''\nonumber \\&\quad + 2\kappa ^2A^2\mathcal {V} = 2\kappa ^2A^2p\,, \end{aligned}$$

116c $$\begin{aligned}&6\mathcal {C}\mathcal {H}' + 6(2\mathcal {C} + \mathcal {A}')\mathcal {H}^2 + 2\mathcal {B}(\Phi '' + 2\mathcal {H}\Phi ')\nonumber \\&\quad + \mathcal {B}'\Phi '^2 + 2\kappa ^2A^2\mathcal {V}' = 0\,. \end{aligned}$$

Note that in these two cases the two functions $\mathcal {K}$ and $\mathcal {L}$ describing the cosmologically symmetric connection do not enter the cosmological dynamics. One finds that the obtained cosmological dynamics agree with those of a class of scalar-torsion theories of gravity for the axial branch of metric teleparallel cosmology and its flat limit [15, 15, 20].

We continue with the spatially curved branch 2b. In this case the metric field equations become

117a $$\begin{aligned}&6\mathcal {A}(\mathcal {H}^2 + u^2) - \mathcal {B}\Phi '^2 + 3(\mathcal {A}' + \mathcal {C})\nonumber \\&\quad \left( \mathcal {L} + \frac{u^2}{\mathcal {L}}\right) \Phi ' - 6\mathcal {C}\mathcal {H}\Phi ' - 2\kappa ^2A^2\mathcal {V} = 2\kappa ^2A^2\rho \,, \end{aligned}$$

117b $$\begin{aligned}&-2\mathcal {A}(2\mathcal {H}' + \mathcal {H}^2 + u^2) + (2\mathcal {C}' - \mathcal {B})\Phi '^2 - 2(2\mathcal {A}' + \mathcal {C})\mathcal {H}\Phi ' \nonumber \\&\quad + (\mathcal {A}' + \mathcal {C})\left( 3\mathcal {L} - \frac{u^2}{\mathcal {L}}\right) \Phi ' + 2\mathcal {C}\Phi '' + 2\kappa ^2A^2\mathcal {V} \nonumber \\&\quad = 2\kappa ^2A^2p\,. \end{aligned}$$

The connection equations are also non-trivial in this case and read

118a $$\begin{aligned}&3(\mathcal {A}' + \mathcal {C})\left( \mathcal {L} + \frac{u^2}{\mathcal {L}}\right) \Phi ' \nonumber \\&\quad = 2\kappa ^2A\left( \omega ' + 3\mathcal {H}\omega + 3\mathcal {L}\chi - 3\frac{u^2}{\mathcal {L}}\psi \right) , \end{aligned}$$

118b $$\begin{aligned}&(\mathcal {A}' + \mathcal {C})\left( \mathcal {L} + \frac{u^2}{\mathcal {L}}\right) \Phi ' \nonumber \\&\quad = 2\kappa ^2A\left( \phi ' + 3\mathcal {H}\phi + \mathcal {L}\chi - \frac{u^2}{\mathcal {L}}\psi \right) \,. \end{aligned}$$

Finally, the scalar equation takes the form

119 $$\begin{aligned}{} & {} 6\mathcal {C}\mathcal {H}' + 6(2\mathcal {C} + \mathcal {A}')\mathcal {H}^2 - 6\mathcal {A}'u^2 - 3(\mathcal {A}' + \mathcal {C})\nonumber \\{} & {} \quad \times \left[ 2\mathcal {H}\left( \mathcal {L} - \frac{u^2}{\mathcal {L}}\right) + \frac{\mathcal {L}'}{\mathcal {L}}\left( \mathcal {L} + \frac{u^2}{\mathcal {L}}\right) \right] \nonumber \\{} & {} \quad + 2\mathcal {B}(\Phi '' + 2\mathcal {H}\Phi ') + \mathcal {B}'\Phi '^2 + 2\kappa ^2A^2\mathcal {V}' = 0. \end{aligned}$$

Before analyzing these equations, we also list the remaining spatially flat branches. In the branch 1c, the field equations become

120a $$\begin{aligned}&6\mathcal {A}(\mathcal {H}^2 + u^2) - \mathcal {B}\Phi '^2 + 3(\mathcal {A}' + \mathcal {C})\mathcal {L} \Phi '\nonumber \\&\quad - 6\mathcal {C}\mathcal {H}\Phi ' - 2\kappa ^2A^2\mathcal {V} = 2\kappa ^2\rho \,, \end{aligned}$$

120b $$\begin{aligned}&\quad -2\mathcal {A}(2\mathcal {H}' + \mathcal {H}^2 + u^2) + (2\mathcal {C}' - \mathcal {B})\Phi '^2 \nonumber \\&\quad - 2(2\mathcal {A}' + \mathcal {C})\mathcal {H}\Phi ' + 3(\mathcal {A}' + \mathcal {C}) \mathcal {L}\Phi ' \nonumber \\&\quad + 2\mathcal {C}\Phi '' + 2\kappa ^2A^2\mathcal {V} = 2\kappa ^2p \end{aligned}$$

120c $$\begin{aligned}&3(\mathcal {A}' + \mathcal {C})\mathcal {L}\Phi ' = 2\kappa ^2A(\omega ' + 3\mathcal {H}\omega + 3\mathcal {L}\chi )\,, \end{aligned}$$

120d $$\begin{aligned}&(\mathcal {A}' + \mathcal {C})\mathcal {L}\Phi ' = 2\kappa ^2A(\phi ' + 3\mathcal {H}\phi + \mathcal {L}\chi )\,, \end{aligned}$$

120e $$\begin{aligned}&6\mathcal {C}\mathcal {H}' + 6(2\mathcal {C} + \mathcal {A}')\mathcal {H}^2 - 6\mathcal {A}'u^2 - 3(\mathcal {A}' + \mathcal {C})(\mathcal {L}' + 2\mathcal {H}\mathcal {L}) \nonumber \\&\quad + 2\mathcal {B}(\Phi '' + 2\mathcal {H}\Phi ') + \mathcal {B}'\Phi '^2 + 2\kappa ^2A^2\mathcal {V}' = 0\,, \end{aligned}$$

while for the branch 1b we find

121a $$\begin{aligned}&6\mathcal {A}(\mathcal {H}^2 + u^2) - \mathcal {B}\Phi '^2 + 3(\mathcal {A}' + \mathcal {C})\mathcal {L}\Phi ' \nonumber \\&\quad - 6\mathcal {C}\mathcal {H}\Phi ' - 2\kappa ^2A^2\mathcal {V} = 2\kappa ^2\rho \,, \end{aligned}$$

121b $$\begin{aligned}&-2\mathcal {A}(2\mathcal {H}' + \mathcal {H}^2 + u^2) + (2\mathcal {C}' - \mathcal {B})\Phi '^2 - 2(2\mathcal {A}' + \mathcal {C})\mathcal {H}\Phi '\nonumber \\&\quad - (\mathcal {A}' + \mathcal {C})\mathcal {L}\Phi '+ 2\mathcal {C}\Phi '' + 2\kappa ^2A^2\mathcal {V} = 2\kappa ^2p \end{aligned}$$

121c $$\begin{aligned}&3(\mathcal {A}' + \mathcal {C})\mathcal {L}\Phi '\nonumber \\&\quad = 2\kappa ^2A(\omega ' + 3\mathcal {H}\omega - 3\mathcal {L}\psi )\,, \end{aligned}$$

121d $$\begin{aligned}&(\mathcal {A}' + \mathcal {C})\mathcal {L}\Phi ' = 2\kappa ^2A(\phi ' + 3\mathcal {H}\phi - \mathcal {L}\psi )\,, \end{aligned}$$

121e $$\begin{aligned}&6\mathcal {C}\mathcal {H}' + 6(2\mathcal {C} + \mathcal {A}')\mathcal {H}^2 - 6\mathcal {A}'u^2 + 3(\mathcal {A}' + \mathcal {C})(\mathcal {L}' + 2\mathcal {H}\mathcal {L})\nonumber \\&\quad + 2\mathcal {B}(\Phi '' + 2\mathcal {H}\Phi ') + \mathcal {B}'\Phi '^2 + 2\kappa ^2A^2\mathcal {V}' = 0\,. \end{aligned}$$

Note that in all three cases only the parameter function $\mathcal {L}$ contributes to the field equations, while $\mathcal {K}$ is absent.

To study the dynamics of the three branches 2b, 1b and 1c, we restrict ourselves to the case of vanishing hypermomentum, and start with the connection equations. For the spatially curved branch 2b, these take the form (118) and can be solved by any of the following solutions:

1. For theories which satisfy the condition $\mathcal {A}' + \mathcal {C} = 0$ one can combine the terms containing $\mathcal {A}$ and $\mathcal {C}$ in the action to  by integrating by parts, and the theory reduces to an equivalent of scalar-curvature gravity [20]. The teleparallel connection variable $\mathcal {L}$ does not appear in the field equations, and the connection equations (118) of motion become trivial, i.e., the left hand side of these equations vanishes, and so they are solved identically for vanishing hypermomentum. The same holds also for the flat branches 1c and 1b.
2. The field equations (118) are also solved if the scalar field is constant, $\Phi ' = 0$. Also in this case $\mathcal {L}$ disappears from the metric field equations (117), and they reduce to the Friedmann equations, up to a constant rescaling of the gravitational constant, mediated by $\mathcal {A}$, which can be absorbed into $\kappa ^2$, as well as a cosmological constant determined by the potential $\mathcal {V}$. The remaining scalar field equation (119), which is a first-order differential equation in $\mathcal {L}$, can then be solved for $\mathcal {L}$. Also these findings apply in full analogy to the flat branches 1c and 1b.
3. For $u^2 < 0$, corresponding to negative spatial curvature, the connection field equations (118) can also be solved by $\mathcal {L} = \pm iu$. Choosing the lower sign, both the metric field equations (117) and the scalar field equation (119) reduce to the dynamics of the vector branch in scalar-torsion gravity [15, 15, 20], while for the upper sign one has a trivial substitution $u \mapsto -u$. The same statement holds also for the spatially flat branches 1c and 1b, where in this case one sets $\mathcal {L} = 0$ and the cosmological dynamics reduce to that of scalar-torsion theory in the limit $u = 0$ of vanishing spatial curvature.

In summary, we find that for all branches of cosmologically symmetric teleparallel geometries the cosmological dynamics of the class of scalar-teleparallel gravity theories defined by the action (110) reduce to those of scalar-torsion gravity [20], or one of its special cases, which are equivalent to scalar-curvature gravity or general relativity with a cosmological constant. In order to obtain a non-trivial contribution also from the nonmetricity to the cosmological dynamics, one needs to study more classes of general scalar-teleparallel theories. We leave such studies for future work.

## Example solution

We have already seen some example solutions of the connection equations of motion in *f*(*G*) gravity in the absence of hypermomentum. Here we want to show another simple case exemplifying effects of the connection and hypermomentum on the metric. We consider the general quadratic gravity in the simple branch 2a. One can show that one can find the exact solution of the connection equations

122 $$\begin{aligned} \mathcal {K}&{=}\frac{\mathcal {K}_0}{A^2}\nonumber \\&\quad {+}\frac{(-3z_6(z_6+2z_7)+4z_8(z_4+z_5))\mathcal {H}+A(4z_8\phi -z_6\omega )}{3z_6^2-4z_4z_8}, \end{aligned}$$

123 $$\begin{aligned} \mathcal {L}&=\frac{\mathcal {L}_0}{A^2} +\frac{(-3z_5z_6+6z_4z_7)\mathcal {H}+A(-3z_6\phi +z_4\omega )}{3z_6^2-4z_4z_8},\nonumber \\ \end{aligned}$$

with $\mathcal {K}_0$ and $\mathcal {L}_0$ integration constants, and we have to assume here $3z_6^2-4z_4z_8\ne 0$. For $3z_6^2-4z_4z_8=0$ only the combination $z_4\mathcal {K}+z_6\mathcal {L}$ appears in the connection equations of motion, so only this sum can be determined from them. We also absorb here $\kappa ^2$ in the hypermomentum variables $\phi$ and $\omega$. Plugging the connection functions in the metric equations of motion yields

124 $$\begin{aligned} 2\kappa ^2\rho&=6\alpha \frac{\mathcal {H}^2}{A^2}+6(z_1+z_2)\frac{u^2}{A^2}\nonumber \\&\quad +\rho _\Gamma +\rho _H+\frac{2}{A}(\omega '+3\mathcal {H}\omega )\,, \end{aligned}$$

125 $$\begin{aligned} 2\kappa ^2 p&=2\alpha (\mathcal {H}'+\mathcal {H}^2)-2(z_1+z_2)\frac{u^2}{A^2}\nonumber \\&\quad +\rho _\Gamma +\rho _H-\frac{2}{A}(\tilde{\phi }'+3\mathcal {H}\tilde{\phi })\,, \end{aligned}$$

with

126 $$\begin{aligned}&\alpha {=}z_1\nonumber \\&\quad {-}\frac{4z_4^2z_8{+}2z_5({-}3z_6^2{-}6z_6z_7{+}2z_5z_8){+}z_4({-}3z_6^2{+}12z_7^2{+}8z_5z_8)}{6z_6^2{-}8z_4z_8}\,, \end{aligned}$$

127 $$\begin{aligned}&\rho _\Gamma = -\frac{3z_4\mathcal {K}_0^2+6z_6\mathcal {K}_0\mathcal {L}_0 +4z_8\mathcal {L}_0^2}{A^6}\,, \end{aligned}$$

128 $$\begin{aligned}&\rho _H =\frac{6\mathcal {K}_0\phi +2\mathcal {L}_0\omega }{A^3} +\frac{12z_8\phi ^2-6z_6\phi \omega +z_4\omega ^2}{3z_6^2-4z_4z_8}\,, \end{aligned}$$

129 $$\begin{aligned}&\tilde{\phi }=\frac{(6z_6z_7-4z_5z_8)\phi +(z_5z_6-2z_4z_7)\omega }{3z_6^2-4z_4z_8}\,. \end{aligned}$$

The constant $\alpha$ determines the coupling to matter, which should be unity to obtain the same coupling as in GR. Spatial curvature again enters as an effective $(z_1+z_2)u^2$. We also find a contribution coming from the connection, $\rho _\Gamma \propto A^{-6}$, which shows that the connection behaves like stiff matter $p_\Gamma =\rho _\Gamma$. Note that the sign of the energy density depends on the signs of the $z_i$, and one can achieve $\rho _\Gamma <0$. Such stiff matter with negative energy density may be used to facilitate a bouncing universe. Hypermomentum enters in a more complicated way.

## Conclusion

We have derived the most general class of homogeneous and isotropic teleparallel geometries, defined by a metric and a flat, affine connection, which are invariant under spatial rotations and translations. We find that there are five branches of such geometries, two of which exhibit a non-vanishing spatial curvature for the metric Levi-Civita connection, while the remaining three spatially flat cases arise as particular limits from the spatially curved cases. We have also shown how these branches are related to the more restricted classes or metric and symmetric teleparallel geometries, in which nonmetricity or torsion are imposed to vanish. Our findings show that in addition to the lapse and scale factor appearing in the homogeneous and isotropic Robertson-Walker metric, the flat affine connection is described by two further functions of time, which may participate in the cosmological dynamics for a suitable gravity theory which couples to these degrees of freedom.

We have then applied our findings to a number of general teleparallel gravity theories and derived their cosmological dynamics. In particular, we have considered the *f*(*G*) class of theories, general teleparallel quadratic gravity and a simple class of scalar-teleparallel gravity theories. We have seen that for the different branches of cosmologically symmetric teleparallel geometries, which are distinct only by the flat, affine connection, one finds, in general, qualitatively different cosmological dynamics, such as a different number of dynamical functions appearing in the cosmological field equations or a different differential order of these equations. Further, our findings show that for the *f*(*G*) and scalar-teleparallel theories the cosmological dynamics fully reduces to that of related metric teleparallel or curvature based gravity theories, in which nonmetricity and possibly torsion are absent, such that the results found for the cosmological dynamics of these simpler theories also apply to their general teleparallel counterparts as follows:

1. Already without imposing cosmological symmetry, *f*(*G*) gravity reduces to general relativity with a cosmological constant (GR$\Lambda$) for $f'' \equiv 0$, while the scalar-teleparallel theories reduce to scalar-curvature gravity (SCG) for $\mathcal {A}' + \mathcal {C} \equiv 0$.
2. In the branch 2a, the dynamics reduces to that of the metric teleparallel counterpart (*f*(*T*) gravity or scalar-torsion gravity (STG)) in the “axial” branch [15], and to its flat limit in the branch 1a. The latter also holds also for vanishing hypermomentum in the branches 1b and 1c for $\mathcal {L} = 0$, while one finds the “vector” branch of metric teleparallel solutions for the branch 2b and $\mathcal {L} = \pm iu$.
3. For the spatially curved branch 2b and the two flat branches 1b and 1c, one finds that if $\mathcal {L}$ does not take the values given in the preceding item, the connection equations with vanishing hypermomentum inevitably yield a solution for which the metric field equations reduce to that of general relativity with a cosmological constant.

It follows that the only possibility to obtain new dynamics in the aforementioned classes of theories is to consider a non-trivial coupling between matter and the teleparallel connection, leading to non-vanishing hypermomentum. For more general theories, new dynamics is obtained. As an explicit example, we have shown this for the class of general teleparallel quadratic gravity theories, but one may also consider theories with a more general dependence of the Lagrangian on the teleparallel geometry or more general scalar field couplings.

Our results allow for several directions of further research. Starting from the homogeneous and isotropic teleparallel geometries we have determined, one can derive and study the cosmological dynamics of further general teleparallel gravity theories. Also among those classes of theories whose cosmological field equations we have derived in this article we find several classes of general teleparallel quadratic gravity theories whose cosmological dynamics qualitatively differs from the previously studied metric and symmetric teleparallel theories, and deserves further attention. Finally, going beyond the cosmological background dynamics one may consider perturbations of the teleparallel geometry around this background and study their dynamics. For a general flat connection one finds that the perturbations are of the form

130 $$\begin{aligned} \delta \Gamma ^\alpha {}_{\mu \nu }=\nabla _\nu \delta \Lambda ^\alpha {}_\mu \, \end{aligned}$$

with $\delta \Lambda ^\alpha {}_\mu$ an arbitrary matrix with 16 entries, in addition to the ten perturbation variables of the metric. Even after gauging away four of these variables one is left with 22 perturbation variables, leading to a much more cumbersome analysis compared to the mere three free functions of the cosmological background metric and connection. An important question to be addressed in these studies is whether the so-called strong coupling problem for linear perturbations around highly symmetric background, which has been found in metric teleparallel gravity theories [19, 21–26], is also present in general teleparallel gravity. FLRW solutions based on non-metricity seem to provide a promising route for this purpose [27]. We leave this question for future investigations.

## Data Availability

This manuscript has no associated data or the data will not be deposited. [Authors’ comment: The work is purely theoretical and no data has been generated or used.]

## Footnotes

1 Note that by the identity  for any vector $V^\mu$, integrating the connection $\nabla$ by parts will introduce additional terms proportional to $M^\mu {}_{\nu \mu }$. We also assume here that boundary terms vanish, e.g., that $\zeta ^\nu$ vanishes on the boundary.
